# Donor *NKG2C* homozygosity contributes to CMV clearance after haploidentical transplantation

**DOI:** 10.1172/jci.insight.149120

**Published:** 2022-02-08

**Authors:** Xing-Xing Yu, Qian-Nan Shang, Xue-Fei Liu, Mei He, Xu-Ying Pei, Xiao-Dong Mo, Meng Lv, Ting-Ting Han, Ming-Rui Huo, Xiao-Su Zhao, Ying-Jun Chang, Yu Wang, Xiao-Hui Zhang, Lan-Ping Xu, Kai-Yan Liu, Xiang-Yu Zhao, Xiao-Jun Huang

**Affiliations:** 1 Peking University People’s Hospital, Peking University Institute of Hematology, National Clinical Research Center for Hematologic Disease, Beijing Key Laboratory of Hematopoietic Stem Cell Transplantation, Beijing, China.; 2 Peking-Tsinghua Center for Life Sciences, Beijing, China.; 3 Beijing Engineering Lab for Cellular Therapy, Beijing, China.

**Keywords:** Transplantation, NK cells

## Abstract

CMV infection remains an important cause of morbidity and mortality after allogeneic hematopoietic stem cell transplantation (allo-HSCT). Several investigators have reported that adaptive NKG2C^+^ NK cells persistently expand during CMV reactivation. In our study, 2 cohorts were enrolled to explore the relationships among the *NKG2C* genotype, NKG2C^+^ NK cell reconstitution, and CMV infection. Multivariate analysis showed that donor *NKG2C* gene deletion was an independent prognostic factor for CMV reactivation and refractory CMV reactivation. Furthermore, adaptive NKG2C^+^ NK cells’ quantitative and qualitative reconstitution, along with their anti-CMV function after transplantation, was significantly lower in patients grafted with *NKG2C^wt/del^* donor cells than in those grafted with *NKG2C^wt/wt^* donor cells. At day 30 after transplantation, quantitative reconstitution of NKG2C^+^ NK cells was significantly lower in patients with treatment-refractory CMV reactivation than in patients without CMV reactivation and those with nonrefractory CMV reactivation. In humanized CMV-infected mice, we found that, compared with those from *NKG2C^wt/del^* donors, adaptive NKG2C^+^ NK cells from *NKG2C^wt/wt^* donors induced earlier and stronger expansion of NKG2C^+^ NK cells as well as earlier and stronger CMV clearance in vivo. In conclusion, donor *NKG2C* homozygosity contributes to CMV clearance by promoting the quantitative and qualitative reconstruction of adaptive NKG2C^+^ NK cells after haploidentical allo-HSCT.

## Graphical abstract

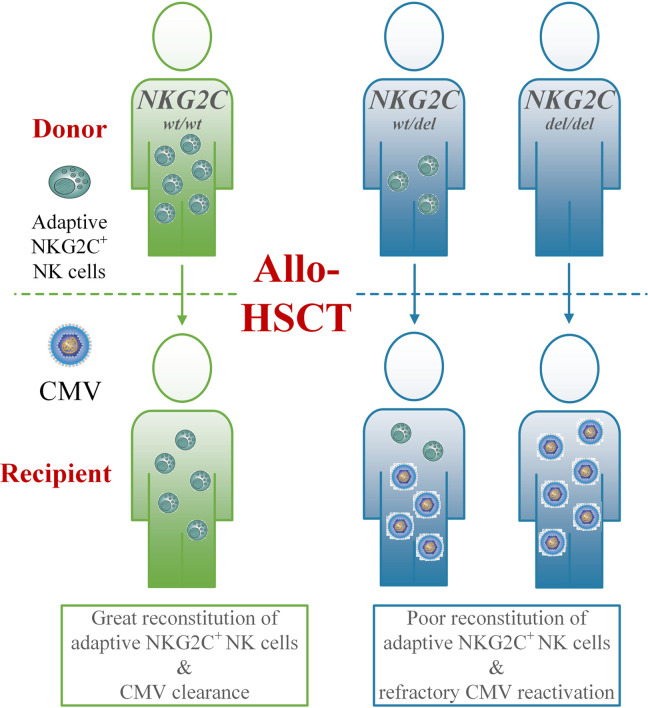

## Introduction

Viral infections, especially CMV infections, remain important causes of morbidity and mortality after allogeneic hematopoietic stem cell transplantation (allo-HSCT) (1, 2). The reconstitution of innate and adaptive immune cells, such as T cells and NK cells, plays an important role in controlling CMV infections (3–7).

Adaptive NKG2C^+^ NK cells are a subgroup of NK cells with high expression of the NKG2C-activating receptor and the absence of its inhibitory counterpart NKG2A (8). One case report showed that in a T cell–deficient infant with acute CMV infection, 80% of the NK cell population expressed NKG2C (9). In vitro data show that NKG2C^hi^CD57^hi^ NK cells are highly responsive to CMV-infected macrophages only in the presence of CMV-specific antibodies; they are functionally poor effectors of natural cytotoxicity in CMV^+^ healthy donors (10). NKG2C receptors contribute to NKG2C^hi^CD57^hi^ NK cell activation, and co–cross-linking of NKG2C enhances the CD16-mediated anti-CMV response (10). In HLA-matched transplantation or umbilical cord blood transplantation, adaptive NKG2C^+^ NK cells can be activated and can proliferate in response to CMV infection (11). In summary, evidence suggests that adaptive NKG2C^+^ NK cells might play a role in controlling CMV infection after transplantation.

Three genotypes of *NKG2C* (i.e., *NKG2C^wt/wt^*, *NKG2C^wt/del^*, and *NKG2C^del/del^*) have been described in humans (12), with frequencies of 63.6%, 32.4%, and 4%, respectively, in Asian and White populations (13, 14). The *NKG2C* genotype influences CD94/NKG2C receptor function, NKG2C surface receptor level, and NKG2C^+^ NK cell number in CMV^+^ individuals, further suggesting the receptor’s active involvement in the CMV-induced reconfiguration of the NK cell compartment (15). Vietzen et al. reported that the heterozygous *NKG2C* genotype is significantly associated with the frequency of CMV viremia and CMV disease after lung transplantation (16). Redondo-Pachón et al. reported that the heterozygous *NKG2C* genotype is associated with symptomatic CMV infection after kidney transplantation (17). Taken together, these studies suggest that a patient’s *NKG2C* genotype is associated with the frequency and severity of CMV infection. However, the mechanisms by which a patient’s *NKG2C* genotype affects CMV infection are still unknown.

Data on the influence of a donor’s *NKG2C* genotype on CMV infection after allo-HSCT are limited. Cao et al. found that after double umbilical cord blood (DUCB) transplantation, the combined graft could contain 0–4 functional copies of the *NKG2C* gene (18). The 6-month cumulative incidence of CMV reactivation was significantly lower in patients who received DUCB grafts with 3 or 4 *NKG2C* copies than in patients who received 2 units with 1 or 2 *NKG2C* copies (18). These authors also reported that CMV reactivation occurs more frequently in patients grafted with *NKG2C^wt/del^* cord blood. Activating KIRs may have a role as, even in the absence of NKG2C expression from donors carrying a homozygous deletion of the *NKG2C* gene, CMV infection can cause rapid NK maturation characterized by the expansion of CD56^dim^NKG2A^–^KIR^+^ cells (19). Therefore, whether a donor’s *NKG2C* genotype affects CMV infection after transplantation merits investigation.

Previous studies reported that the quantity of adaptive NKG2C^+^ NK cells is associated with the *NKG2C* genotype in healthy individuals (20). The 3 genotypes of donor-derived NK cells provide an excellent platform for exploring the impact of the donor *NKG2C* genotype on NKG2C^+^ NK cell reconstitution and CMV infection after allogeneic stem cell transplantation. Therefore, we sought to investigate whether donor *NKG2C* genotype affects the reconstitution of adaptive NKG2C^+^ NK cells and whether their reconstitution contributes to the clearance of CMV infection.

## Results

### Donor NKG2C homozygosity is associated with CMV clearance after allo-HSCT.

**Figure 1:**
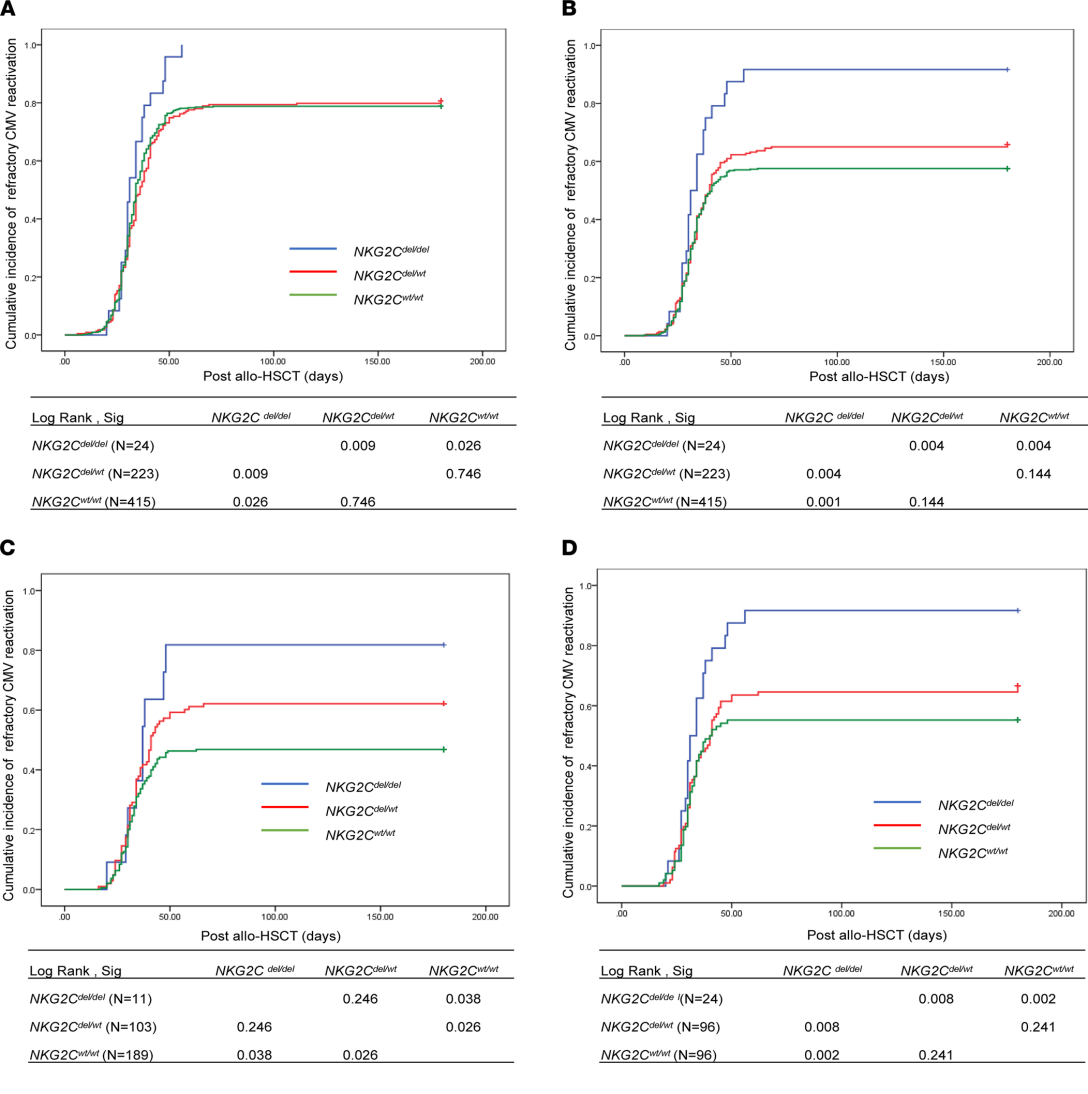
Cumulative incidence of CMV reactivation and refractory CMV reactivation among 3 *NKG2C* genotypes. Kaplan-Meier curves of the cumulative incidences of (**A**) CMV reactivation and (**B**) refractory CMV reactivation stratified by *NKG2C^del/del^* (*n* = 24), *NKG2C^wt/del^* (*n* = 223), and *NKG2C^wt/del^* (*n* = 415) in the first cohort. (**C**) Kaplan-Meier curves showing cumulative incidence of refractory CMV reactivation among patients without aGVHD stratified by *NKG2C^del/del^* (*n* = 11), *NKG2C^wt/del^* (*n* = 103), and *NKG2C^wt/del^* (*n* = 189) in the first cohort. (**D**) Kaplan-Meier curves for cumulative incidence of refractory CMV reactivation in the first cohort stratified by *NKG2C^del/del^* (*n* = 24), *NKG2C^wt/del^* (*n* = 96), and *NKG2C^wt/del^* (*n* = 96). Log-rank (Mantel-Cox) was used for these statistics.

There were 662 patients in the first of this study’s 2 cohorts. Table 1 presents a detailed analysis of the association between the *NKG2C* deletion genotype and CMV reactivation. There were 24 patients with the donor genotype *NKG2C^del/del^*, 223 with genotype *NKG2C^wt/del^*, and 415 with *NKG2^wt/wt^*. The occurrence of CMV reactivation increased significantly (*P* = 0.039) with decreasing presence of the donor *NKG2C* gene (78.8% for *NKG2C^wt/wt^*, 80.7% for *NKG2C^wt/del^*, and 100.0% for *NKG2C^del/del^*). As shown in Figure 1A, the cumulative incidence of CMV reactivation after transplantation was significantly higher in the group with the *NKG2C^del/del^* genotype.

Of the 24 patients with *NKG2C^del/del^*, 22 showed refractory CMV reactivation. The occurrence of refractory CMV reactivation increased significantly (*P* = 0.001) with decreasing presence of the donor *NKG2C* gene (57.6% for *NKG2C^wt/wt^*, 65.9% for *NKG2C^wt/del^*, and 91.7% for *NKG2C^del/del^*). As shown in Figure 1B, the cumulative incidence of refractory CMV reactivation after transplantation was significantly higher in the group with the *NKG2C^del/del^* genotype.

Among patients without acute graft-versus-host disease (aGVHD), refractory CMV reactivation increased significantly (*P* = 0.007) with decreasing presence of the donor *NKG2C* gene (46.8% for *NKG2C^wt/wt^*, 62.1% for *NKG2C^wt/del^*, and 81.8% for *NKG2C^del/del^*). The cumulative incidence of refractory CMV reactivation after transplantation was significantly higher in the group with the *NKG2C^del/del^* genotype (Figure 1C). Among patients with aGVHD, those with full (100%) deletion of the *NKG2C* gene showed significantly higher refractory CMV reactivation (*P* = 0.041) than those with *NKG2C^wt/del^* (69.2%) or *NKG2C^wt/wt^* (66.7%). However, the difference was less significant between patients with the *NKG2C^wt/del^* donor genotype and those with the *NKG2C^wt/wt^* donor genotype.

We analyzed other variables that may affect CMV reactivation or refractory CMV reactivation, including patient age, donor age, donor type, incidence of aGVHD, and donor/recipient serology. Factors with *P* values of less than or equal to 0.05 in univariate analyses were included in the subsequent Cox multivariate analysis. As Table 2 shows, the *NKG2C* genotype remained significantly associated with CMV reactivation and refractory CMV reactivation (*P* = 0.048 and *P* = 0.004, respectively).

Among the 662 patients in the first cohort, 17 were diagnosed with CMV disease. There were 8 patients with CMV pneumonia, 6 with CMV enteritis, and 3 with CMV retinitis. Because of the small number of patients in this subset, the cumulative incidence of CMV disease (0.0%, 2.7%, 2.7%) was significantly comparable (*P* = 0.723) among the donor *NKG2C* genotypes (*NKG2C^wt/wt^*, *NKG2C^wt/del^*, and *NKG2C^del/del^*).

A total of 60 patients died of transplantation-related mortality (TRM); 88 patients relapsed after transplantation, and 61 died of relapse. No significant differences were found in the incidences of relapse or of TRM, leukemia-free survival, or overall survival among the patient groups with different donor genotypes of the *NKG2C* gene (*NKG2C^wt/wt^*, *NKG2C^wt/del^*, *NKG2C^del/del^*; data not shown).

To control for other factors, patients in the *NKG2C^del/del^* group were matched at a ratio of 1:4 (by age, sex, primary disease, blood type, HLA mismatch number, and incidence of aGVHD) with patients in the *NKG2C^wt/del^* and *NKG2C^wt/wt^* groups (Supplemental Table 1; supplemental material available online with this article; https://doi.org/10.1172/jci.insight.149120DS1). According to the Cox multivariate analysis, the *NKG2C* genotype remained significantly associated with CMV reactivation and refractory CMV reactivation (*P* = 0.045 and *P* = 0.004, respectively; Supplemental Table 2). The cumulative incidence of refractory CMV reactivation after transplantation was significantly higher in the group with the *NKG2C^del/del^* genotype (Figure 1D). No significant differences were found in incidences of CMV disease, relapse, TRM, leukemia-free survival, or overall survival among patients with differing donor genotypes of the *NKG2C* gene (*NKG2C^wt/wt^, NKG2C^wt/del^, NKG2C^del/del^*; data not shown).

### Donor NKG2C deletion affects the quantitative and qualitative reconstitution of adaptive NKG2C^+^ NK cells after allo-HSCT.

**Figure 2:**
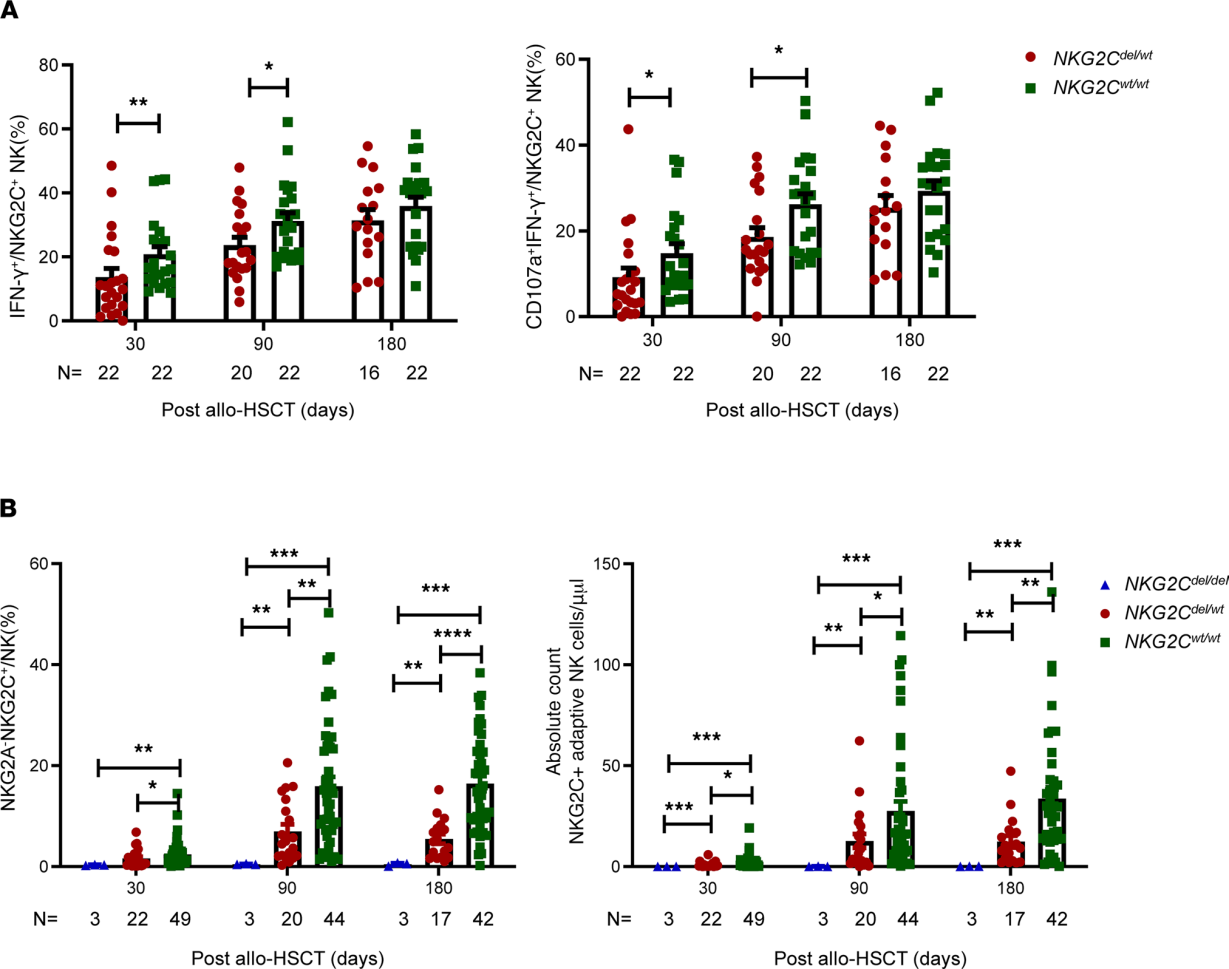
Quantitative and qualitative reconstitution of NKG2C^+^ adaptive NK cells in different *NKG2C*-genotype groups. (**A**) Percentages of CD107a^+^ and CD107a^+^IFN-γ^+^ NKG2A^–^NKG2C^+^ NK cells among K562 cells in the *NKG2C^wt/del^* and *NKG2C^wt/wt^* groups. Data are expressed as the mean and SEM. (**B**) Percentage and absolute count of NKG2A^–^NKG2C^+^ NK cells among the *NKG2C^del/del^*, *NKG2C^wt/del^*, and *NKG2C^wt/wt^* groups at days 30, 90, and 180 after allo-HSCT. *P < 0.05, **P < 0.01, ***P < 0.001, ****P < 0.0001. We performed (**A**) a nonparametric *t* test with Mann-Whitney and (**B**) a 1-way ANOVA test.

To investigate the impact of a donor’s *NKG2C* genotype on the reconstitution of adaptive NKG2C^+^ NK cells after transplantation, we prospectively enrolled a continuous cohort of patients who underwent haploidentical transplantation. As shown in Figure 2, patients with the donor *NKG2C^del/del^* genotype had no reconstitution of adaptive NKG2C^+^ NK cells. We found that the percentage of adaptive NKG2C^+^ NK cells among the total NK cell population was significantly lower in the *NKG2C^wt/del^* group than in the *NKG2C^wt/wt^* group at days 30 (1.615% ± 0.3837% vs. 2.442% ± 0.3707%, *P* = 0.0395), 90 (6.988% ± 1.367% vs. 15.94% ± 1.846%, *P* = 0.0045), and 180 (5.491% ± 0.9548% vs. 16.43% ± 1.508%, *P* < 0.0001) after transplantation. The absolute cell counts of adaptive NKG2C^+^ NK cells were also significantly lower in the *NKG2C^wt/del^* group than in the *NKG2C^wt/wt^* group at days 30 (0.9422 ± 0.2866 cells/μl vs. 2.174 ± 0.4572 cells/μl, *P* = 0.0231), 90 (12.73 ± 3.440 cells/μl vs. 27.55 ± 4.800 cells/μl, *P* = 0.0493), and 180 (12.41 ± 2.949 cells/μl vs. 33.65 ± 4.582 cells/μl, *P* = 0.0047) after transplantation. Notably, donor *NKG2C* deletion had no effect on the reconstitution of other NK cell subsets after transplantation (Supplemental Figure 1).

To further investigate functional reconstitution in the *NKG2C^wt/wt^* and *NKG2C^wt/del^* groups, we prospectively analyzed stored patient specimens. Patients in the *NKG2C^wt/del^* group served as a control group, matched at a 1:1 ratio (by age, sex, primary disease, blood group, number of HLA mismatches, and incidence of aGVHD) with patients in the *NKG2C^wt/wt^* group (Supplemental Table 3). Figure 2 shows that the percentage of IFN-γ^+^ adaptive NKG2C^+^ NK cells among K562 cells was significantly lower in the *NKG2C^wt/del^* group than in the *NKG2C^wt/wt^* group at days 30 (13.69% ± 2.742% vs. 20.79% ± 2.388%, *P* = 0.0079) and 90 (23.68% ± 2.488% vs. 31.22% ± 2.548%, *P* = 0.0268) after transplantation. The percentage of IFN-γ^+^CD107a^+^ adaptive NKG2C^+^ NK cells among the total adaptive NKG2C^+^ NK cell population was significantly lower in the *NKG2C^wt/del^* group than in the *NKG2C^wt/wt^* group at days 30 (9.160% ± 2.160% vs. 14.73% ± 2.180%, *P* = 0.0223) and 90 (18.56% ± 2.193% vs. 26.24% ± 2.383%, *P* = 0.0465) after transplantation. At day 180 after transplantation, the functional reconstitution of NKG2C^+^ NK cells against K562 cells showed no differences between the 2 groups. There were no significant differences between the 2 groups in the expression levels of CD122, DNAM-1, NKP30, NKP46, and CD25 in adaptive NKG2C^+^ NK cells (Supplemental Figure 2).

**Figure 3:**
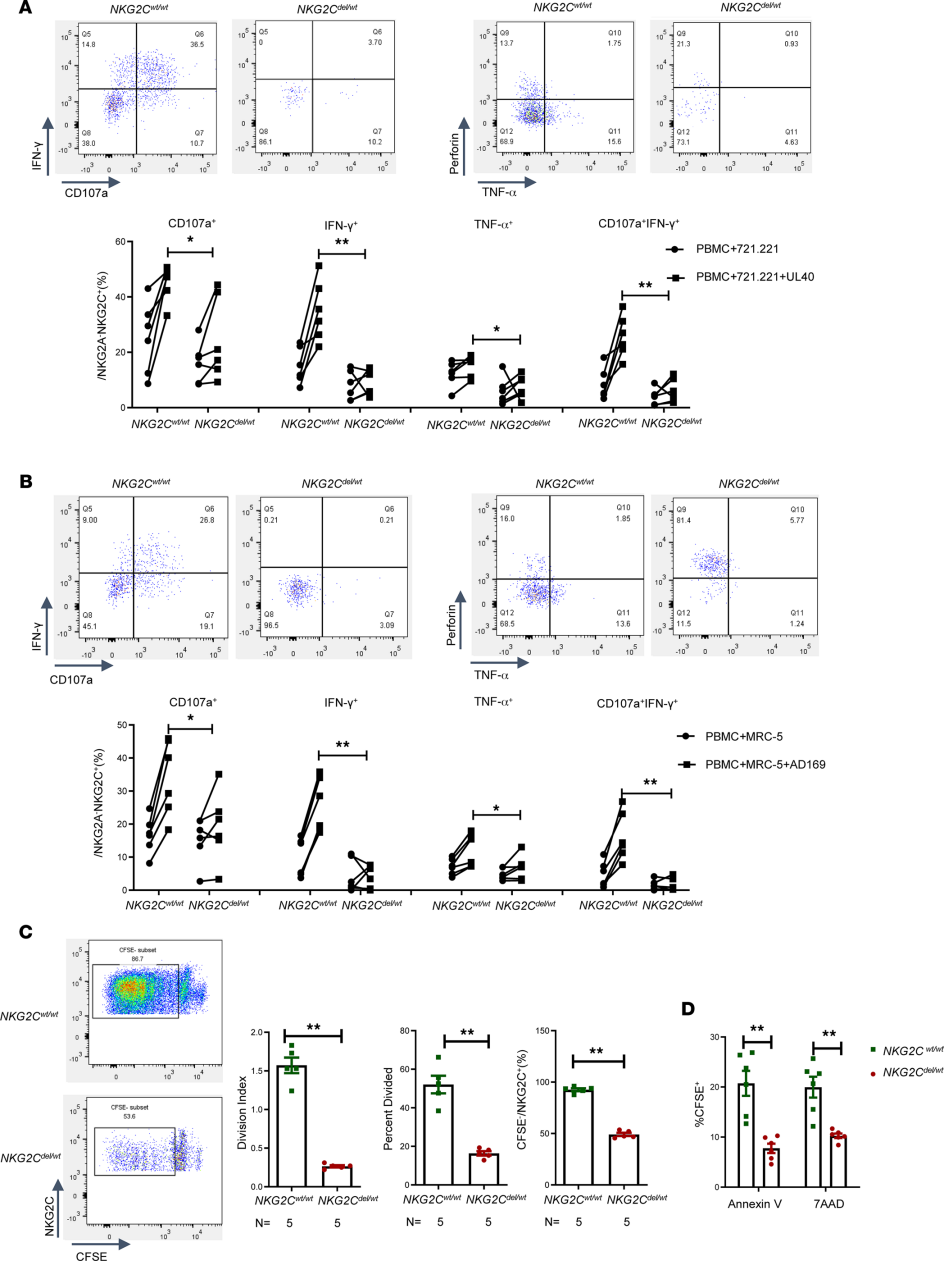
Anti-CMV functional reconstitution of NKG2C^+^ adaptive NK cells in different *NKG2C*-genotype groups. PBMCs from patients who had CMV reactivation at day 30 after transplantation were cocultured with 721.221 or UL40-721.221 cells, or with MRC-5 or AD169-MRC-5 cells. Representative images of the expression of CD107a^+^, IFN-γ^+^, TNF-α^+^, and Perforin^+^ on NKG2A^–^NKG2C^+^ NK cells in the *NKG2C^wt/wt^* group and *NKG2C^wt/del^* group. (**A**) Coculture with 721.221 or UL40-721.221 cells, (**B**) MRC-5 or AD169-MRC-5 cells, with percentages of CD107a^+^, IFN-γ^+^, TNF-α^+^, Perforin^+^ and CD107a^+^IFN-γ^+^ NKG2A^–^NKG2C^+^ NK cells. (**C**) CFSE-labeled sorted donor NK cells were cocultured or not with UL40-721.221 cells. Representative images taken seven days after expression of CFSE^–^NKG2A^–^NKG2C^+^ NK cells in the *NKG2C^wt/wt^* group and *NKG2C^wt/del^* group, with percentage of CFSE^–^NKG2A^–^NKG2C^+^ NK cells, division index, and percent divided in the *NKG2C^wt/wt^* and *NKG2C^wt/del^* groups. (**D**) NKG2C^+^ NK cells were isolated from donor NK cells using NKG2C^+^ positive beads. Then, CFSE-labeled AD169-MRC-5 cells were used as targets at an effector-to-target ratio of 10:1 for 5 hours. Data are expressed as the mean and SEM. *P < 0.05, **P < 0.01. Nonparametric Student’s *t* test with Mann-Whitney.

To detect the anti-CMV function of adaptive NKG2C^+^ NK cells from patients with different donor *NKG2C* genotypes in vitro, the cytotoxicity of each patient’s NKG2C^+^ NK cells was evaluated against AD169-MRC-5 cells and UL40-721.221 cells. All patients showed CMV reactivation at 30 days after transplantation (*n* = 12). In the cocultures with UL40-721.221 cells, the percentages of CD107a^+^ (45.15% ± 2.634% vs. 24.59% ± 6.071%, *P* = 0.0152), IFN-γ^+^ (34.94% ± 4.420% vs. 9.133% ± 1.881%, *P* = 0.0022), TNF-α^+^ (15.12% ± 1.553% vs. 7.615% ± 1.699%, *P* = 0.0260), and CD107a^+^IFN-γ^+^ (25.72% ± 3.045% vs. 6.107% ± 1.757%, *P* = 0.0022) cells among NKG2A^–^NKG2C^+^ NK cells were significantly higher in the *NKG2C^wt/wt^* group than in the *NKG2C^wt/del^* group. This pattern was also observed in the cocultures with AD169-MRC-5 cells, the percentages of CD107a^+^ (34.02% ± 4.662% vs. 19.20% ± 4.304%, *P* = 0.0411), IFN-γ^+^ (25.72% ± 3.315% vs. 4.225% ± 1.407%, *P* = 0.0022), TNF-α^+^ (12.06% ± 2.009% vs. 6.877% ± 1.485%, *P* = 0.0368), and CD107a^+^IFN-γ^+^ (16.15% ± 2.977% vs. 1.535% ± 0.8312%, *P* = 0.0022) cells being significantly higher in the *NKG2C^wt/wt^* group than in the *NKG2C^wt/del^* group. Regarding the proliferation of adaptive NKG2C^+^ NK cells in donors, the percentage of CFSE^–^ (92.56% ± 1.394% vs. 49.08% ± 1.567%, *P* = 0.0079) cells among NKG2A^–^NKG2C^+^ NK cells was significantly higher in the *NKG2C^wt/wt^* group. The proliferation index (1.572 ± 0.1013 vs. 0.2640 ± 0.01470, *P* = 0.0079) and the percent divided (52.08% ± 4.582% vs. 16.36% ± 1.129%, *P* = 0.0079) were also significantly higher in the *NKG2C^wt/wt^* group. Furthermore, the apoptosis rate of AD169-MRC-5 cells was significantly higher in the *NKG2C^wt/wt^* group (Annexin V: 20.73% ± 2.500% vs. 7.752% ± 0.9382%, *P* = 0.0022; 7AAD: 19.97% ± 2.091% vs. 10.27% ± 0.4488%, *P* = 0.0022). These results indicate that donor *NKG2C* deletion damages the anti-CMV function of donor-derived adaptive NKG2C^+^ NK cells after transplantation (Figure 3).

### Delays in quantitative reconstitution of adaptive NKG2C^+^ NK cells foster the occurrence of refractory CMV reactivation.

In the second cohort, 65 patients experienced CMV reactivation, with a median time to reactivation of 34 days after transplantation. Among these patients, 37 experienced refractory CMV reactivation.

**Figure 4:**
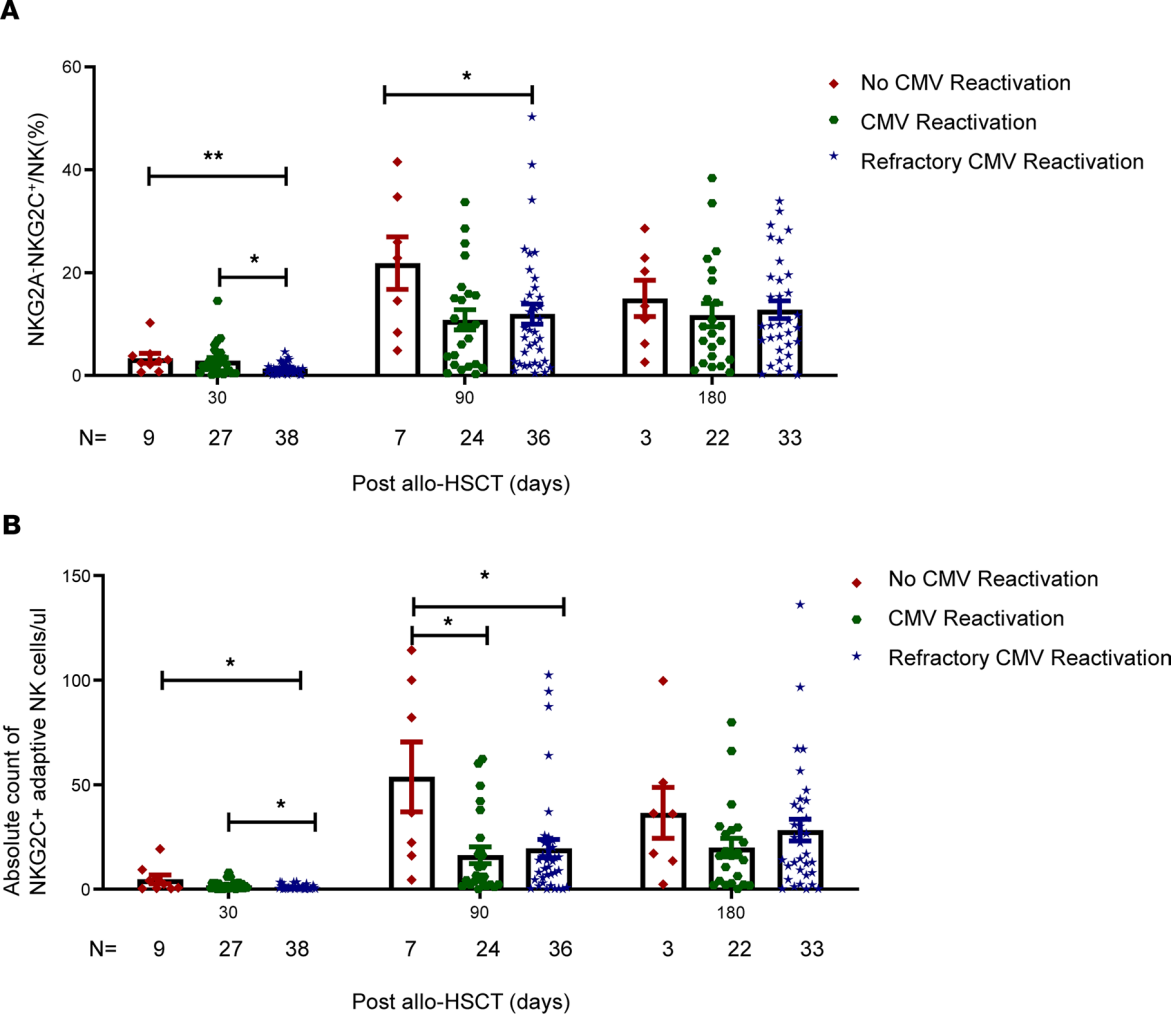
Poor quantitative reconstitution of NKG2C^+^ adaptive NK cells in patients with refractory CMV reactivation at day 30 after allo-HSCT. (**A**) Percentage of NKG2A^–^NKG2C^+^ NK cells in the no-CMV-reactivation, CMV-reactivation, and refractory-CMV-reactivation groups at days 30, 90, and 180 after allo-HSCT. (**B**) Absolute count of NKG2C^+^ NK cells in the no-CMV-reactivation, CMV-reactivation, and refractory-CMV-reactivation groups at days 30, 90, and 180 after allo-HSCT. Data are expressed as the mean and SEM. *P < 0.05, **P < 0.01. One-way ANOVA.

We divided the patients into 3 groups according to reactivation status: no CMV reactivation, CMV reactivation, and refractory CMV reactivation. As shown in Figure 4, at day 30 after transplantation and before CMV reactivation detection, we found that the percentage of NKG2C^+^ NK cells was significantly lower in the refractory CMV group than in the no-CMV-reactivation group (1.285 ± 0.1761% vs. 3.334 ± 0.9537%, *P* = 0.0108) and in the CMV-reactivation group (1.285 ± 0.1761% vs. 2.865 ± 0.5941%, *P* = 0.0177). In addition, the absolute cell counts of NKG2C^+^ NK cells in the refractory CMV group were significantly lower than those in the no-CMV-reactivation (0.8915 ± 0.1652 vs. 4.716 ± 2.080 cells/μl, *P* = 0.0206) and CMV-reactivation groups (0.8915 ± 0.1652 vs. 1.889 ± 2.681 cells/μl, *P* = 0.0461). These differences among the 3 groups gradually disappeared from day 90 to day 180 after transplantation.

**Figure 5:**
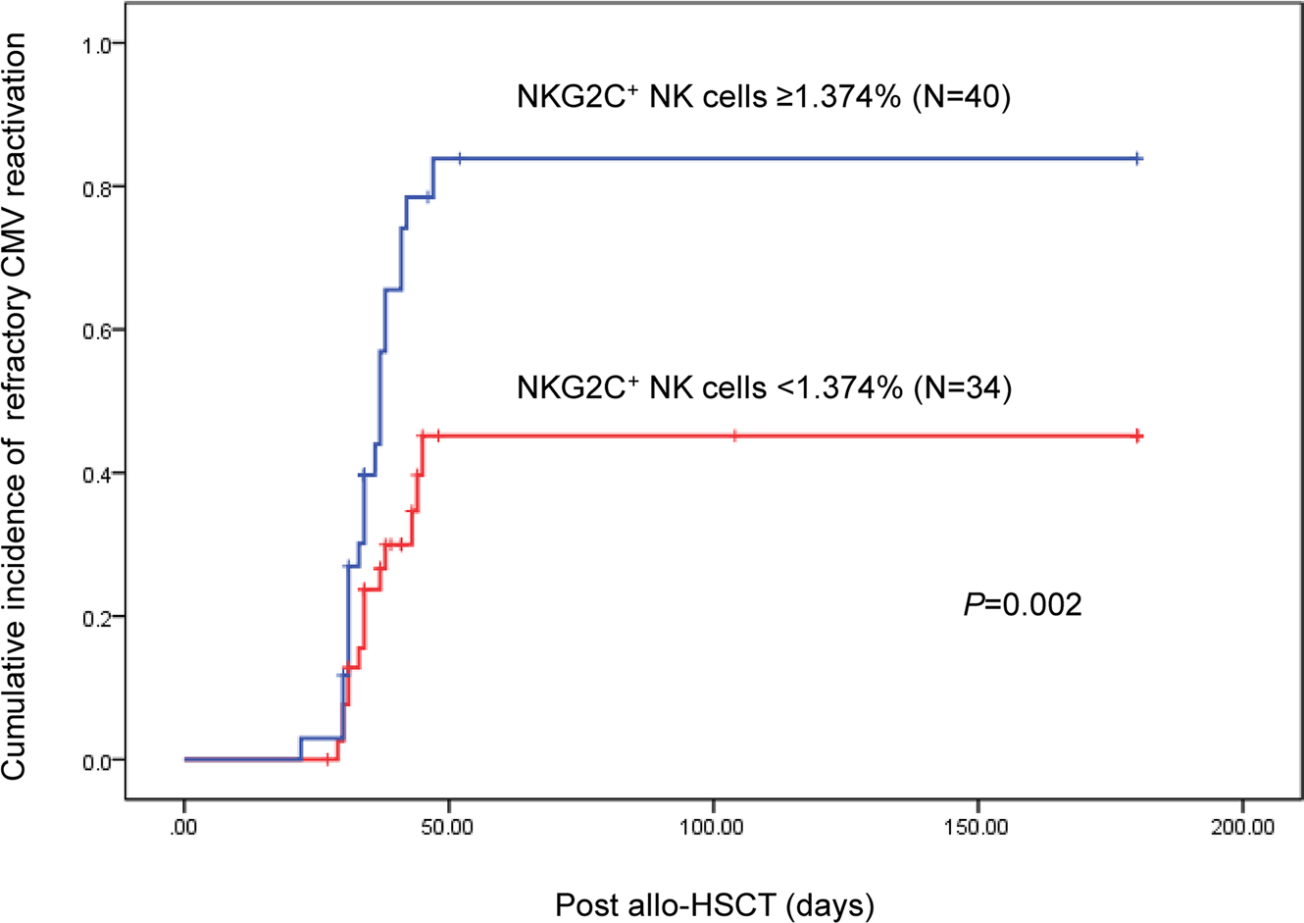
Refractory CMV reactivation associated with reconstitution of NKG2C^+^ adaptive NK cells at day 30 after allo-HSCT. Kaplan-Meier curves for cumulative incidence of refractory CMV reactivation stratified by percentage of NKG2A^–^NKG2C^+^ NK cells after allo-HSCT. A log-rank (Mantel-Cox) test was used for these statistics.

At day 30 after transplantation, based on the receiver operating characteristic curve, we calculated a cutoff to define the percentage of NKG2C^+^ NK cells that best discriminated between higher and lower risk for refractory CMV reactivation (1.374%). The incidence of refractory CMV reactivation was markedly lower in the group with a higher percentage of NKG2C^+^ cells (14 of 40) than in the group with a lower percentage (23 of 34). In addition, as shown in Figure 5, the cumulative incidence of refractory CMV reactivation after transplantation was significantly lower in patients with higher percentages of NKG2C^+^ NK cells (35% vs. 67.7%, *P* = 0.005). Using the Cox regression model, we found that a percentage of NKG2C^+^ NK cells < 1.374% at day 30 was a risk factor for the occurrence of refractory CMV reactivation (HR = 0.407, 0.209–0.794, *P* = 0.008). This result demonstrated that the quantitative reconstitution of adaptive NKG2C^+^ NK cells was associated with the occurrence of refractory CMV activation.

### Correlation between the reconstitution of pentamer^+^ CMV-specific CD8 T cells (CMV-CTLs) and that of adaptive NKG2C^+^ NK cells after transplantation.

In vitro experiments demonstrated that NKG2C^+^ NK cells can regulate the expansion of CMV-CTLs (21); however, the correlation between the reconstitution of NKG2C^+^ NK cells and CMV-CTLs after transplantation was unknown. We therefore collected peripheral blood (PB) from 10 patients who had HLA-A*0201^+^ or HLA-A*-2402^+^ donors before viral reactivation. The samples were collected at aGVHD onset after hematopoietic cell transplantation (HCT), at diagnosis of CMV, and at 2, 4, and 8 weeks after antiviral therapy to monitor the reconstitution of pentamer^+^ CD8 T cells (22) as well as NKG2C^+^ NK cells. In these 10 patients, we found that the percentage of NKG2C^+^ NK cells and the absolute number of NKG2C^+^ NK cells were correlated with the absolute number of CMV-CTLs after transplantation (Supplemental Figure 3, A and B). Reconstitution of CMV-CTL was delayed compared to NKG2C^+^ NK cell reconstitution at different time points after CMV infection (Supplemental Figure 3, C and D). However, because of the limited number of patients, we did not find differences in the reconstitution of CMV-CTLs between the *NKG2C^wt/wt^* group and *NKG2C^wt/del^* group (data not shown).

### Adaptive NKG2C^+^ NK cells from NKG2C homozygous donors show a stronger ability to clear CMV infection in a humanized mouse model.

**Figure 6:**
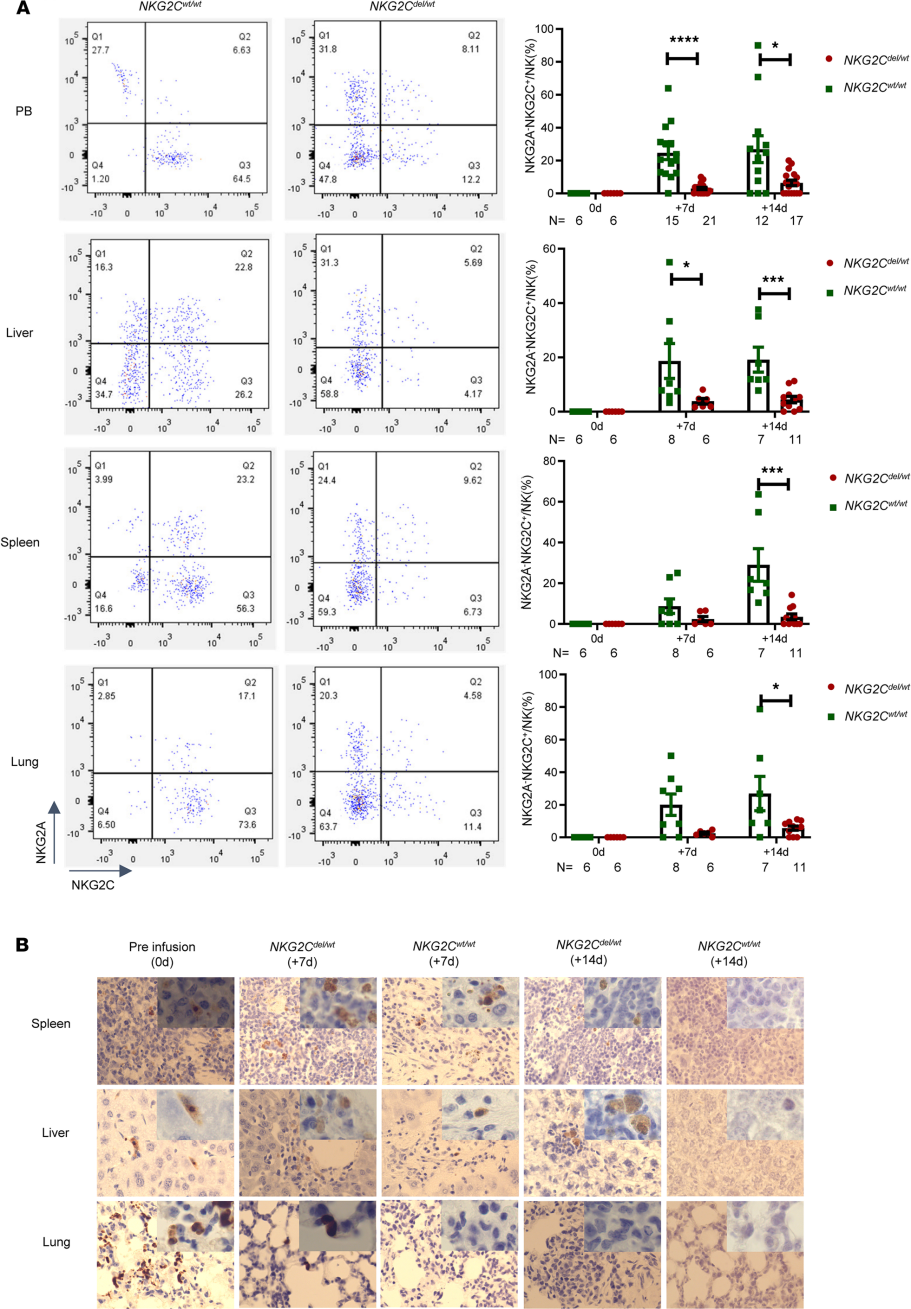
Stronger CMV clearance ability of *NKG2C^wt/wt^* adaptive NKG2C^+^ NK cells over other types of NKG2C^+^ NK cells in a humanized mouse model. (**A**) Representative images taken at day 14 after infusion of the expression of NKG2A^–^NKG2C^+^ NK cells in the *NKG2C^wt/wt^* group and *NKG2C^wt/del^* group. The percentages of NKG2A^–^NKG2C^+^ NK cells in PB, spleen, liver, and lung in the *NKG2C^wt/wt^* group and *NKG2C^wt/del^* group at days 0, 7, and 14. (**B**) CMV pathology in the spleen, liver, and lung of animals in the *NKG2C^wt/wt^* and *NKG2C^wt/del^* groups at days 0, 7, and 14 (original magnification, ×40 and ×100). At least 5 mice were evaluated at each time point. Data are expressed as the mean and SEM. *P < 0.05, **P < 0.01, ***P < 0.001, ****P < 0.0001. Nonparametric Student’s *t* test with Mann-Whitney.

To detect whether adaptive NKG2C^+^ NK cells from different *NKG2C* genotypes exhibited different expansion and CMV clearance capabilities in vivo, we established a CMV-infected humanized mouse model. There were no significant differences in aGVHD or in percentages of NK cells and NKG2A^+^ NK cells after NK cell infusion (Supplemental Figure 4). The genotypes of 6 donors in humanized mice were determined to exclude the influence of ADCC among different donors; this was described in the Supplemental Results and Supplemental Table 4. As shown in Figure 6, adoptively transferred NK cells had the ability to migrate to the spleen, liver, and lungs and persisted in these target organs for 14 days. In PB (26.89% ± 8.154% vs. 6.409% ± 1.662%, *P* = 0.0166), liver (19.13% ± 4.587% vs. 4.458% ± 1.161%, *P* = 0.0003), spleen (29.00% ± 8.001% vs. 3.546% ± 1.444%, *P* = 0.0001), and lungs (26.91% ± 10.52% vs. 5.647% ± 1.256%, *P* = 0.0359), the percentage of NKG2A^–^NKG2C^+^ NK cells was significantly higher in the *NKG2C^wt/wt^* group than in the *NKG2C^wt/del^* group (Figure 6). These findings indicated that the proliferation of adaptive NKG2C^+^ NK cells was significantly higher in the *NKG2C^wt/wt^* group than in the *NKG2C^wt/del^* group. At day 14 after infusion we also found that *NKG2C^wt/wt^* adaptive NKG2C^+^ NK cells effectively fought systemic CMV infection by diminishing CMV-typical lesions in target organs (Figure 6).

In contrast, mice in the *NKG2C^wt/del^* NK cell infusion group remained positive for CMV DNA in the spleen and liver but showed CMV clearance in the lungs. This finding suggested that in our mouse model, adaptive NKG2C^+^ NK cells from *NKG2C^wt/wt^* donors exert stronger anti-CMV function compared with those from other donors. We then investigated the effect of CMV-IgG on enhanced CMV clearance in mice in the *NKG2C^wt/del^* NK cell infusion group. As Supplemental Figure 8A shows, on day 14 after NK infusion, mice in the IgG group remained positive for CMV DNA in the spleen, liver, and lung. Mice in the NK plus IgG injection group showed CMV clearance only in the lung and no difference from mice in the NK infusion group. Moreover, the migration of NK cells showed no significant difference between mice with CMV-IgG injection and those without (Supplemental Figure 8B). Therefore, CMV-IgG showed no effect on enhanced CMV clearance by adaptive NK cells.

## Discussion

We found that donor *NKG2C* homozygosity affected CMV clearance by promoting the quantitative and qualitative reconstitution of adaptive NKG2C^+^ NK cells after haploidentical transplantation of hematopoietic stem cells.

We found that the *NKG2C* genotype affects the occurrence of refractory CMV reactivation, defined as CMV reactivation lasting over 2 weeks after transplantation. A study of kidney transplantation showed trends toward increased *NKG2C^wt/del^* and reduced *NKG2C^wt/wt^* frequencies associated with symptomatic CMV infection in patients monitored for CMV viremia every 2 weeks (17); these findings are consistent with our results. Another study found that patients with the *NKG2C^wt/del^* and *NKG2C^deldel^* genotypes have an increased risk of HIV infection and disease progression (23); this was the first indication that the *NKG2C* genotype is associated with virus control. Our research supported this theory by describing a correlation between donor *NKG2C* genotype and CMV control after transplantation. Multivariate analysis and case-pair analysis revealed that donor *NKG2C* genotype was a risk factor for refractory CMV reactivation after transplantation. Vietzen et al. reported that the *NKG2C^wt/wt^* genotype is significantly more frequent than the *NKG2C^wt/del^* genotype in nonviremic patients than in viremic patients or in patients with CMV disease after lung transplantation (16). Our multivariate analysis supported the univariate analysis reported by Vietzen et al. Moreover, our humanized CMV mouse model showed that the *NKG2C^wt/wt^* genotype was associated with better function of adaptive NKG2C^+^ NK cells in reducing the incidence of CMV infection in vivo. In patients who received DUCB, owing to a limited number of patients, Cao et al. found only a trend toward increased CMV reactivation frequency associated with *NKG2C^wt/del^* grafted cord (18). Our research supplied additional evidence of a stable link between the *NKG2C* genotype and the control of CMV infection.

We found that donor *NKG2C* homozygosity promotes quantitative and qualitative reconstitution of adaptive NKG2C^+^ NK cells but not of other NK cell subsets. Further investigation showed that donor *NKG2C* genotype determined the anti-CMV function and proliferation of adaptive NKG2C^+^ NK cells in patients experiencing CMV infection after transplantation. We also found that poor quantitative reconstitution of adaptive NKG2C^+^ NK cells at +30 days affected the clearance of CMV infection. These data supported the hypothesis that poor reconstitution of adaptive NKG2C^+^ NK cells would lead to the incidence of refractory CMV infection. Based on these findings and the qualitative reconstitution data, we speculate that the functions of adaptive NKG2C^+^ NK cells influenced the clearance of CMV infection.

Furthermore, we found that adaptive NKG2C^+^ NK cells can be studied in a humanized mouse model, and our study of CMV-infected humanized mice yielded direct evidence that adaptive NKG2C^+^ NK cells can be activated and can proliferate in response to CMV stimulation. These data contributed information on the mechanism of CMV clearance, suggesting new directions for treating clinical CMV infection and disease.

Consistent with our hypothesis, adaptive NKG2C^+^ NK cells from *NKG2C* homozygous donors showed stronger CMV clearance in cohort 2. While previous studies showed that adaptive NKG2C^+^ NK cells expand during CMV infection (11, 24), we found evidence that, in humanized mice with CMV, donor *NKG2C* deletion damages the anti-CMV function of adaptive NKG2C^+^ NK cells in vivo. Since the ability of adaptive NK cells is also influenced by HLA-E (01 or 03 alleles), IgG1 (G1m3 or G1m17) and CD16a (CD16A-V/V, CD16A-V/F or CD16A-F/F) polymorphisms, we determined the genotypes of 6 donors in humanized mice (data are shown in the supplemental material). The results showed that HLA-E IgG1 and CD16a exhibited a balanced distribution between NKG2C wild-type and heterozygous donors. The interaction of HLA-E (01 vs. 03 alleles), CMV IgG1, CD16a polymorphism, and the *NKG2C* genotype did not have a significant effect on the ability of adaptive NK cells; this finding calls for further exploration.

In sum, we found that donor *NKG2C* homozygosity was beneficial to the reconstitution and anti-CMV function of adaptive NKG2C^+^ NK cells.

Several studies have reported that adaptive NK cells help prevent relapse in HLA-matched HCT. It has been proposed that adaptive NK cells expand preferentially in reduced-intensity recipients or HLA-matched sibling transplantation settings after CMV reactivation, and a direct association has been found between these cells’ expansion and a lower rate of leukemia relapse (25). However, the reactive rate of CMV in haploidentical allo-HSCT was more than 80%, significantly higher than that in HLA-matched HCT. Because adaptive NK cells’ expansion in response to CMV reactivation was more common in haploidentical allo-HSCT, it was difficult to determine the effect of adaptive NK cell reconstitution on relapse.

On the other hand, studies have revealed stronger graft-versus-leukemia effects with haploidentical allografts than with HLA-matched stem cell transplantation (26, 27). Compared with MHC matching, haplomatching of leukemia cells’ MHCs with recipient mouse T cells prolongs leukemic mouse survival, mainly via reduced T cell apoptosis and enhanced secretion of T cell cytokines including IFN-γ and TNF-α (26). Compared with HLA-matched HCT, our therapy strategy of allo-HSCT without T cell depletion in vitro significantly decreased leukemia relapse with the strong effects of T cells, so the association between adaptive NK cell expansion and relapse may have weakened. Such weakening might explain the lack of an observed effect in our cohort.

In conclusion, by promoting the quantitative and qualitative reconstitution of adaptive NKG2C^+^ NK cells, donor *NKG2C* homozygosity contributes to the clearance of CMV infection after haploidentical allo-HSCT. Thus, considering CMV infection risk in haploidentical allo-HSCT, *NKG2C* homozygous donors may be preferable for hematopoietic transplantation. However, our results must be confirmed in HLA-matched sibling or unrelated allo-HSCT settings.

## Methods

### Patients.

There were 2 patient cohorts. The first and larger cohort initially comprised 854 patients who received haploidentical transplantation between June 2012 and December 2019 for acute myeloid leukemia, chronic myeloid leukemia, or myelodysplastic syndrome. We excluded 192 patients from analysis: 29 who underwent prophylactic donor lymphocyte infusion, 21 who died without CMV infection within 100 days after transplantation, and 142 for whom *NKG2C* genotyping data were lacking. Finally, 662 patients were analyzed to measure the effect of donor *NKG2C* genotype on CMV reactivation after transplantation. These patients’ characteristics are shown in Table 1.

To investigate the reconstitution of adaptive NKG2C^+^ NK cells, we analyzed a prospective cohort of 74 consecutive patients with malignant hematologic disorders who underwent haploidentical transplantation at Peking University People’s Hospital between May 2016 and April 2017. Recipients and donors were CMV seropositive before transplantation. The major characteristics of these patients are shown in Table 3. Patient blood was analyzed for quantitative and qualitative reconstitution of NK cells at days 30, 90, and 180 after transplantation. To exclude the impact of CMV reactivation on 30-day adaptive NKG2C^+^ NK reconstitution, patients with CMV reactivation before day 30 were excluded from the cohort. We monitored the patients by measuring twice a week for CMV reactivation, performing quantitative PCR in the clinical virology laboratory. PB from 10 patients whose donors were HLA-A*0201^+^ or HLA-A*2402^+^ was collected before viral reactivation (at the onset of aGVHD), at diagnosis of CMV, and 2, 4, and 8 weeks after antiviral therapy to monitor the reconstitution of CD8 pentamer T cells (22) and NKG2C^+^ NK cells.

### Transplants.

All patients received myeloablative conditioning (28, 29). The transplant procedure was performed as previously described (28, 29). The conditioning regimen for haploidentical transplantation was modified BUCY+ATG (thymoglobulin) consisting of cytarabine (4 g/m^2^ per day) intravenously on days –10 to –9; busulfan (3.2 mg/kg per day) intravenously on days –8 to –6; cyclophosphamide (CY, 1.8 g/m^2^ per day) intravenously on days –5 to –4; Me-CCNU (250 mg/m^2^) orally once on day –3; and thymoglobulin (ATG, Sang Stat; 2.5 mg/kg per day) intravenously for 4 consecutive days from day –5 to –2. All participants received fresh granulocyte colony-stimulating factor (G-CSF)–mobilized BM and PB cells. Ganciclovir was administered during conditioning (through day –2), and acyclovir (400 mg twice a day) was given until the discontinuation of all immunosuppressive agents. Patients also received prophylactic drugs to prevent infection by fungi.

### Monitoring for CMV reactivation, preemptive therapy, and definition.

After transplantation, CMV reactivation was monitored twice weekly using plasma CMV DNA testing with real-time PCR kits from PG Biotech Co., Ltd.

All patients received prophylactic acyclovir from day 1 to day 30 and ganciclovir from day –10 to day –2. Preemptive therapy with either intravenous ganciclovir or intravenous foscarnet was given when PCR results were positive for > 600 copies/mL CMV in two consecutive tests or >1000 copies/mL CMV in a single test on PB, as previously reported (31). Treatment was given for 2 weeks at the full dose and maintained for another 2 weeks until CMV DNA was cleared. Continued treatment consisted of a combination of foscarnet (60 mg/kg intravenously, twice daily) and immunoglobulin when CMV became refractory (1, 30–33).

CMV infection was diagnosed at 2 consecutive counts of CMV DNA ≥ 1 × 10^3^ copies/mL in PB as determined by CMV PCR assay. Refractory reactivation was defined as CMV reactivation lasting over 2 weeks (31, 32, 34).

### NKG2C genotyping.

Donor DNA was isolated from total blood using the Puregene BloodCore kit B (Qiagen). *NKG2C* zygosity was assessed as previously described (12), and the patients were divided into *NKG2C^del/del^*, *NKG2C^wt/wt^*, and *NKG2C^wt/del^* groups according to donor genotype.

### Cell lines.

We purchased MHC class I-deficient human erythron-leukemia cell line K562 and MRC-5 human fetal lung fibroblasts from the National Infrastructure of Cell Line Resource. Xiongwen Wu (Huazhong University of Science and Technology, Department of Immunology, Wuhan, China) provided HLA-I–deficient EBV-transformed B lymphoblastoid cell line 721.221. These cell lines had been authenticated within the last 3 years using STR profiling. All experiments were performed using mycoplasma-free cells.

### Cell culture and virus infection.

The 721.221 and K562 cell lines were cultured in RPMI 1640 (Sigma-Aldrich) complete medium. MRC-5 cells were cultured in DMEM (Sigma-Aldrich) complete medium supplemented with 1% nonessential amino acids (Sigma-Aldrich).

The CMV wild-type strain AD169 (Shanghai Medial College, Fudan University) was propagated using MRC-5 cells. For the preparation of purified CMV stocks, cells were infected at a low MOI (ca. 0.05), and supernatants were harvested when the cells showed strong cytopathic effects. After the removal of cell debris by centrifugation, the supernatants were centrifuged at 20,000*g* for 3 hours at 10°C. The pellets were resuspended in PBS, dounced, and centrifuged through a 20% sorbitol cushion at 65,000*g* for 1 hour at 10°C. Each virus pellet was resuspended in PBS and stored at –80°C. Virus titers were determined using standard plaque assays (35).

### Flow cytometric analyses.

Acquisition was performed on an LSRFortessa instrument (BD Biosciences). Analysis was performed using FlowJo software (TreeStar; see supplemental data for antibody details).

### Cytotoxicity assays.

Cytotoxicity assays were performed using PBMCs and K562 cells as previously described by Zhao et al. (36).

### Anti-CMV functional assays.

MRC-5 fibroblasts were seeded into 24-well plates (1 × 10^5^ per well) and grown in complete medium. Once they reached confluency, they were either infected with AD169 at an MOI of 2 for 2 hours (AD169-MRC-5) or left untreated. Cells were then incubated at 37°C in a 5% CO_2_ humidified incubator and monitored daily for the development of cytopathic effects (24 hours).

Then, 300 μM synthetic CMV *UL40*-encoded peptides (VMAPRTLFL; GL Biochem) was added to 721.221 cells (UL40-721.221) as target cells cultured at a density of 2 × 10^6^ cells/mL in serum-free Opti-MEM (Thermo Fisher) and incubated for 16 hours at 37°C (37).

Before coculture, PBMCs were cultured in RPMI 1640 medium with 10% fetal calf serum and 1000 IU/mL IL-2 for 10–14 hours (38).

For the cytotoxicity assay, MRC-5, AD169-MRC-5, 721.221, and UL40-721.221 cells were used as targets at an effector-to-target ratio of 5:1 for 5 hours. GolgiStop (0.7 μl/mL, BD Biosciences) was added 1 hour after initiation of the assay.

For the apoptosis assay, AD169-MRC-5 cells were labeled with CFSE (BD Horizon) and used as targets at an effector-to-target ratio of 10:1 for 5 hours. NKG2C^+^ NK cells were isolated from NK cells using NKG2C^+^ positive beads (MACS, REA205). After coculture, AD169-MRC-5 cells were analyzed using an Annexin V and 7-amino-actinomycin D apoptosis detection kit (Becton Dickinson) according to the manufacturer’s instructions.

### In vitro proliferation of NK cells.

PBMCs were obtained from healthy donors, and NK cells were sorted using an NK Cell Isolation Kit (Miltenyi Biotec) according to the manufacturer’s recommendations. Enriched NK cells, which included > 90%–95% CD56^+^CD3^–^ cells (data not shown), were treated with IL-15 (10 ng/mL) in the presence or absence of the irradiated UL40-pulsed 721.221 cell line. Purified NK cells were stained with CFSE and monitored for 7 days to examine their proliferation ability. The division index and proliferation index were calculated by the FlowJo Proliferation Platform. The division index is the average number of cell divisions that a cell in the original population has undergone. The calculation includes cells that never divided. The proliferation index is the total number of divisions divided by the number of cells that initiated division. The proliferation index only considers the cells that underwent at least one division; that is, only responding cells are reflected in the proliferation index.

### CMV-infected humanized mice and intravenous injection of in vitro–expanded NK cells.

Fresh PBMCs (2 × 10^7^ cells) were incubated in a T75 culture flask with equal numbers of irradiated mbIL21/41BBL-K562 cells and 1000 IU/mL human IL-2 in RPMI 1640 medium supplemented with autologous serum. The medium was replaced daily with fresh medium and IL-2. One week after expansion, the NK cells were transferred into T175 culture flasks. At two weeks after expansion, the expanded NK cells were transferred into a culture bag. At the beginning of the expansion process, PBMCs were stimulated once with K562-mbIL21-41BBL feeder cells.

Six- to eight-week-old NOD-Prkdc^scid^ IL2Rγc^null^ mice (NPG) were obtained from the Nanjing Model Animal Center. They were sublethally irradiated (150 cGy X-irradiation), and G-CSF–mobilized PBSCs (1 × 10^6^) obtained from CMV-seropositive donors at 0 weeks were injected via the tail vein. At 2 weeks after engraftment, mice were injected i.p. with 1 × 10^6^ AD169-MRC-5 cells (39, 40). At 4 weeks after engraftment, mice received adoptive transfers of in vitro-expanded NK cells (1 × 10^7^) and were injected i.p. with 50,000 U of IL-2 every other day after NK cell infusion. To investigate the effect of CMV-IgG on CMV clearance, mice were injected with CMV-IgG (40 U; Taibang) i.p. twice a week. To assess the expansion of NK cells, mice were killed at days 0, 7, and 14 after NK cell infusion. The spleen, liver, and lungs were harvested and analyzed for CMV DNA by in situ hybridization using a CMV probe (BOND CMV Probe, PB0614). Mice received adoptive transfers of in vitro–expanded NK cells from 6 healthy donors, 3 individual donors from each genetic group (*NKG2C^wt/del^*, *NKG2C^wt/wt^*), and transfer was repeated 3 times. More than 5 mice per group were harvested at each time point.

### Statistics.

Differences in categorical variables between groups were evaluated using χ^2^ tests or Fisher’s exact test. Continuous variables were compared using a nonparametric test (Mann-Whitney test for 2 groups and Kruskal-Wallis test for multiple groups). Correlations were analyzed via nonparametric correlation analysis. The associations among the *NKG2C* genotype, reconstitution of adaptive NKG2C^+^ NK cells, and refractory CMV reactivation were analyzed with a cumulative incidence curve in a competing risk setting, with nonrelapse mortality as a competing event. Multivariate Cox proportional hazards models were constructed to assess the proportional hazards assumption and to test interaction terms with covariates. Statistical analyses were performed using SPSS 19.0, GraphPad Prism 6.0 and R software. *P* values of less than 0.05 were considered statistically significant.

### Study approval.

All patients and donors provided written informed consent, and the Institutional Review Board of the Peking University Institute of Hematology approved the study (ClinicalTrials.gov NCT02978274). Mouse experiments were performed in accordance with the Animal Welfare Act and approved by the Peking University People’s Hospital Institutional Animal Care and Use Committee.

## Author contributions

XXY conducted the in vitro experiments and flow cytometry assays. QNS conducted the animal experiments and performed the statistical analyses; XFL and MH facilitated in vitro and animal experiments; XYP, XDM, ML, TTH, MRH, XSZ, YJC, YW, XHZ, LPX, and KYL performed the clinical examination; XJH and XYZ designed the study and interpreted the data; XJH, XYZ, and XXY wrote the manuscript. QNS revised the manuscript. XXY is listed before QNS because of XXY’s role in writing and manuscript and QNS’s role in revising the manuscript. All authors read and approved the final manuscript.

## Supplementary Material

Supplemental data

## Figures and Tables

**Table 3:**
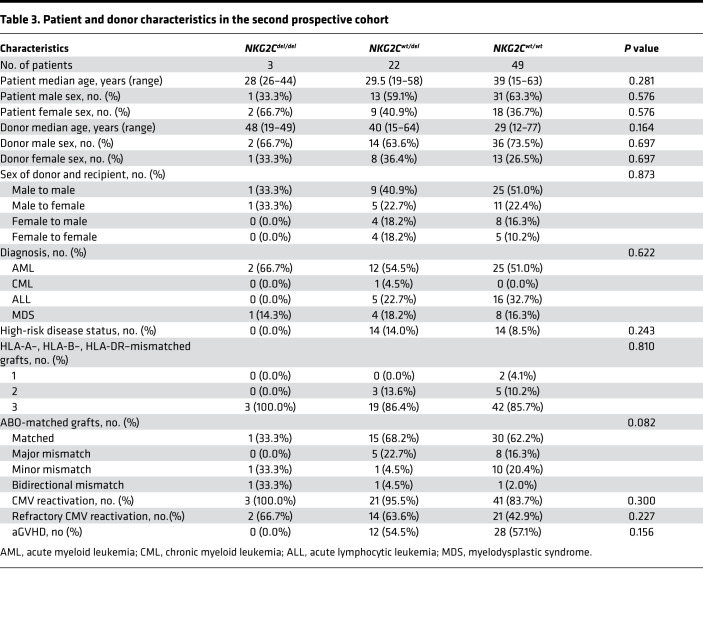
Patient and donor characteristics in the second prospective cohort

**Table 2:**
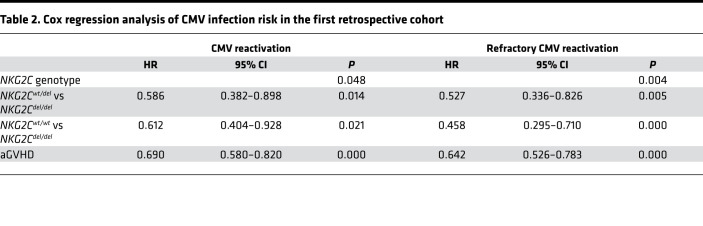
Cox regression analysis of CMV infection risk in the first retrospective cohort

**Table 1:**
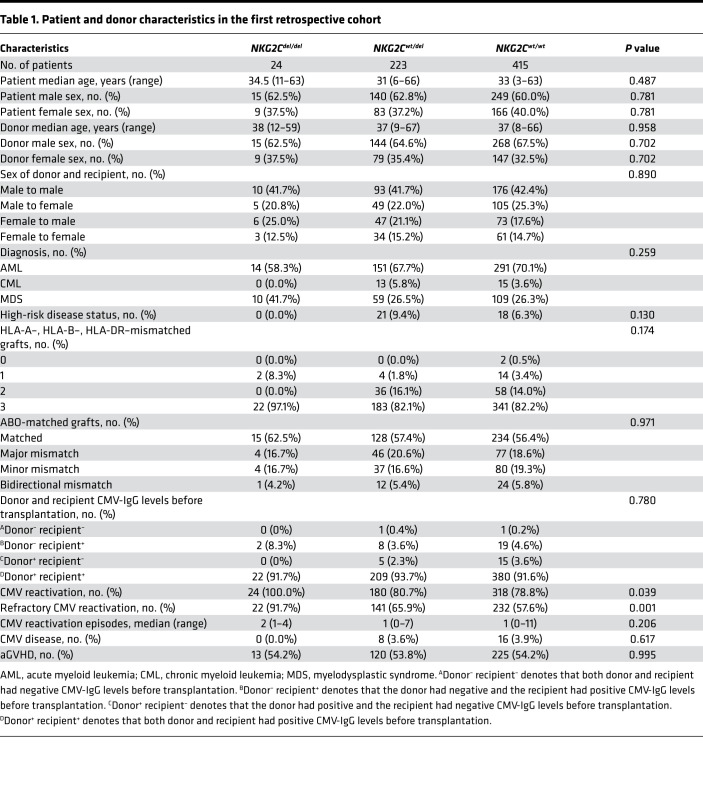
Patient and donor characteristics in the first retrospective cohort

## Version 1 01/06/2022

In-Press Preview

## Version 2 02/08/2022

Electronic publication
