# Supplementary material for: Donor NKG2C homozygosity contributes to CMV clearance after haploidentical transplantation
Source: JCI Insight. 2022 Feb 8;7(3):e149120. doi: 10.1172/jci.insight.149120 (PMC8855817; doi:10.1172/jci.insight.149120)
Supplement: Supplemental data [file jciinsight-7-149120-s264.pdf]

Donor *NKG2C* homozygosity contributes to CMV clearance after haploidentical transplantation

Supplementary Materials

Methods

Flow cytometric analyses

Surface marker staining for the following markers was performed in PBS at 4°C for 30 mins following Fc block: CD3 (BD, UCHT1), CD56 (BD, NKCM16), CD335 (NKp46) (BD, 9E2/NKp46), CD337 (NKp30) (BD, p30-15), CD57 (BD, NK-1), DNAM1(BD, DX11), NKG2C (CD159c) (Milteny Biotec, REA205), NKG2A (CD159a) (Milteny Biotec, REA110), NKG2D (BD, 1D11), CD158a (BD, HP-3E4), CD158b (BECMAN COULTER), CD158e (Milteny Biotec, REA168), Bcl-2 (BD, Bcl-2/100), CD25 (BD, 2A3), CD122(BD, Mik-β3), DNAM-1(BD, DX11). CD107a (BD, H4A3), IFN-γ (Biolegend, B27), TNF-α (BD, MAb11), Granzyme B (BD, GB11), Perforin (Biolegend, B-D48), FcεRI (Milli-Mark, polyclonal), SYK (Invitrogen, 4D10.1).

IgG1, CD16, and HLA-E genotyping

IgG1 markers G1m3 and Glm17 (a G-to-A substitution determining an arginine-to-lysine change at residue 214 of the CH1 region) were determined as previously described (1). We used a TaqMan genotyping assay from Applied Biosystems (Foster City, CA). In brief, the assay included PCR with primers 5’-CCCAGACCTACATCTGCAACGTGA-3’ (forward) and 5’-CTGCCCTG-GACTGGGACTGCAT-3’ (reverse), which specifically amplify a 161-bp fragment of the IGHG1 gene, as well as probes that discriminate the single nucleotide polymorphisms VIC-CTCTCACCAACTTTCTTGT-NFQ (G1m17 specific) and FAM-CTCTCACCAACTCTCTTGT-NFQ (G1m3 specific). CD16a was determined as CD16A-V and CD16A-F (valine/phenylalanine-158 allotypes with different IgG

affinities) by the SNP assay. Briefly, the assay included PCR with primers 5’-GTGATGGTGATGTTCACAGTCTCT-3’ (forward) and 5’-CCAAAAGCCACACTCAAAGAC-3’ (reverse), which specifically amplify a 95-bp fragment of the FCGR3A gene, as well as probes that discriminate the single nucleotide polymorphisms VIC- CTCCCAACAAGCC-NFQ (CD16A-V specific) and FAM-TCCCAAAAAGCC-NFQ (CD16A-F specific).

HLA-E of 6 donors in humanized mice was genotyped by sequence-based typing (SBT) (2). PCR amplification included the primers 5′-GGGGTCGGGATGGAAACGGC-3′ (forward) and 5′-TGAGGTCTGTCAGCTGTGGG-3′ (reverse) to amplify exons 1-7 (3.4 kb). A initial denaturation step was performed at 96°C for 90 seconds and was followed by 35 cycles of 95°C for 30 seconds, 62°C for 60 seconds, and 72°C for 2 minutes and a final extension step at 15°C. The PCR products were purified, and exons 1-5 were sequenced using an ABI Prism Big Dye Terminator 3.1 Cycle Sequencing Kit (Applied Biosystems, California) in an ABI Prism 3130 Genetic Analyzer (Applied Biosystems, California).

Establishment of a mouse model for assessing the antileukemia effect of expanded NK cell products

To test the ability of expanded NK cell products to control leukemia in vivo, we engrafted K562-luciferase-expressing (K562-luc) leukemia cells into NPG mice (6 to 8 weeks old) at day 0 and adoptively transferred 2-week-expanded NK cell products to the mice. In all, 1×10^6 K562-luc+ cells in PBS (200 μL/mouse) were injected via the tail vein on day 0. NK cell products (1×10^7) from the same donor were injected via the

tail vein on day 1. NK cell survival was maintained via intraperitoneal injection of 50,000 IU rhIL-2 every other day for 3 weeks. PB was assessed continuously until no NK cell products could be detected.

Tumor progression in NPG mice was monitored using tumor-derived photons. For imaging, mice were intraperitoneally injected with D-luciferin (15 ng/ml) (Yeasen, Shanghai, China) in PBS and imaged on a Lumia II system (PerkinElmer, USA). Bioluminescence imaging was performed at the indicated time points.

Results

HLA-E, IgG1 and CD16a exhibited balanced distributions between the *NKG2C*$^{wt/wt}$ and *NKG2C*$^{wt/del}$ groups of humanized mice

Since the ability of adaptive NK cells is influenced by HLA-E (01 or 03 alleles), IgG1 (G1m3 or G1m17) and CD16a (CD16A-V/V, CD16A-V/F or CD16A-F/F) polymorphisms, we determined the genotypes of 6 donors in humanized mice (Table). As previously reported, FCGR3A dimorphism results in CD16A-valine/phenylalanine-158 allotypes with different IgG affinities. The CD16A-158V/V allotype of CD16a and the G1m3 allotype of IgG1 enhanced NK cell-mediated Ab-dependent cellular cytotoxicity (ADCC). IgG1 aggregates carrying the G1m17 allotype bind with higher affinity to CD16A-158V/V NK cells than to CD16A-158F/F NK cells(1). For HLA-E, high surface expression of the HLA-E*01:01 allele can enhance the inhibition of NK cells via NKG2A/HLA-E interactions, which in turn attenuate the ADCC of NK cells(3). As shown in Supplementary Table 4, HLA-E IgG1 and CD16a exhibited balanced distributions between NKG2C wild-type and heterozygous donors. These results demonstrated that the NKG2C genotype plays important roles in determining the ability of adaptive NK cells. The interaction effect of HLA-E (01 vs. 03 alleles), CMV IgG1, CD16a polymorphisms and NKG2C genotype on the ability of adaptive NK cells warrants further exploration.

Supplementary Figure 1

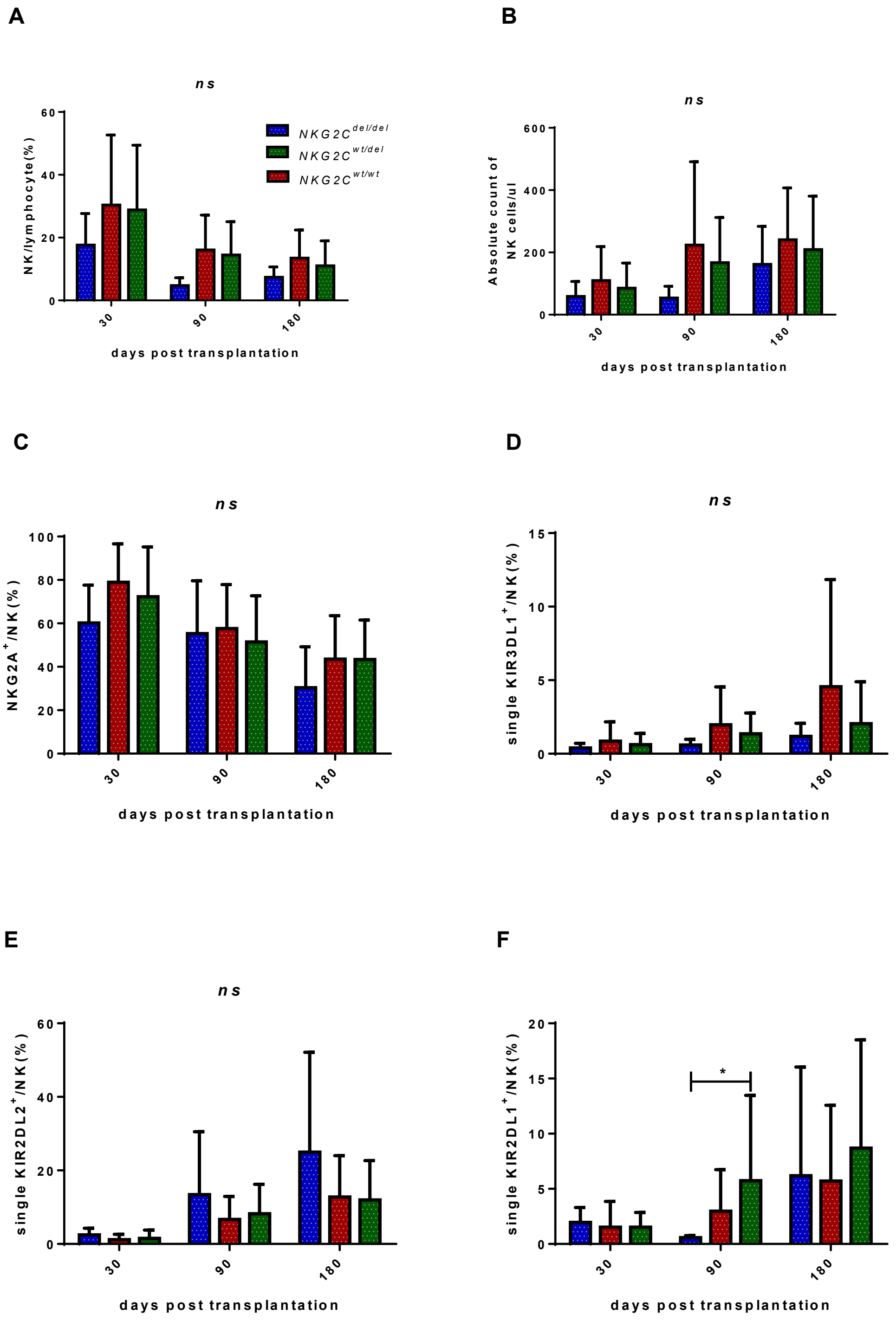

Supplementary Figure 1. Quantitative reconstitution of different NK cell subsets in three *NKG2C*-genotype groups after allo-HSCT. Data are expressed as the mean and SEM. *, $p<0.05$.

Supplementary Figure 2

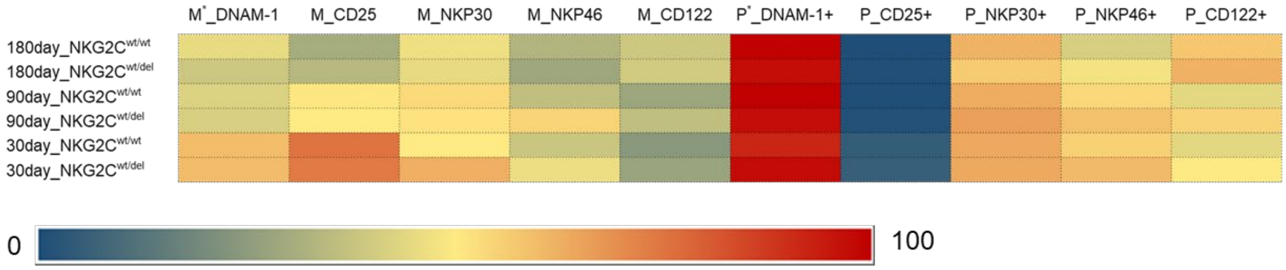

Supplementary Figure 2. The expression of surface markers on adaptive NKG2C+ NK cells in three *NKG2C*-genotype groups after allo-HSCT. Expression was visualized as heatmaps using the heatmap function of R. To transform all the data to the same order of magnitude, we processed the data as follows: the MFI of DNAM-1 was divided by 100; the MFI of NKP30, NKP46 and CD122 was divided by 10; and the expression of CD25 was multiplied by 10.

Supplementary Figure 3

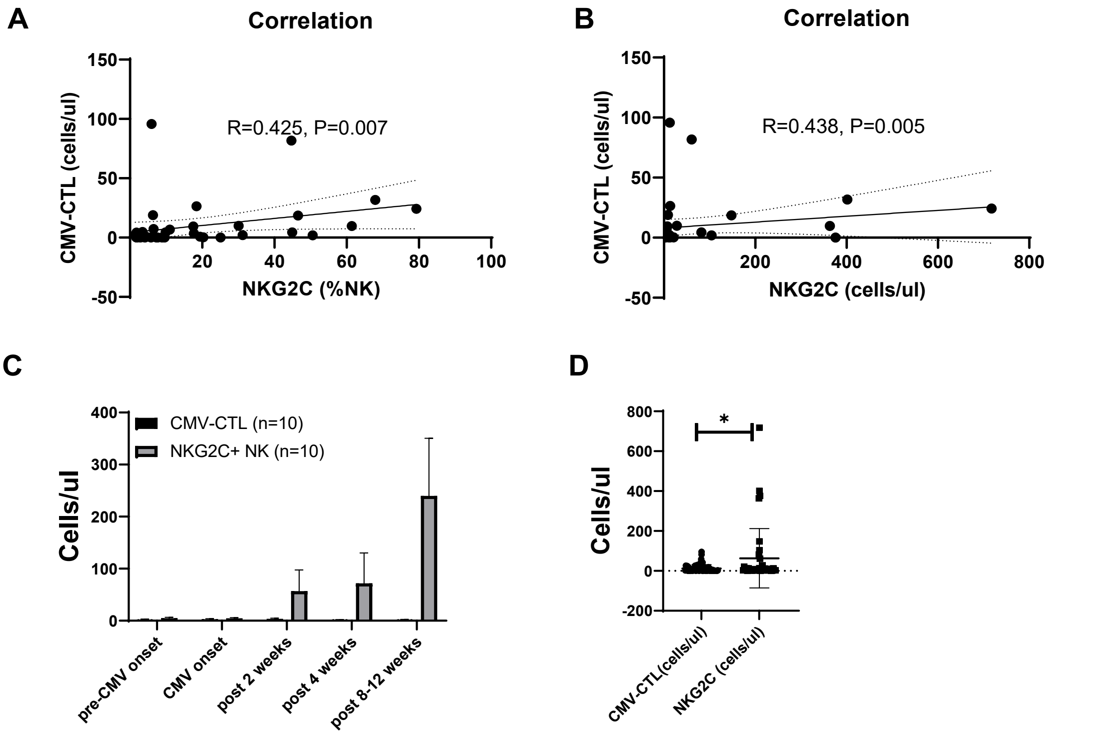

Supplementary Figure 3. The correlation between reconstitution of the percentage of NKG2C+ NK cells in NK cells (A) or the absolute number of NKG2C+ NK cells (B) and the absolute number of CMV-CTLs post transplantation. Comparisons between the absolute number of NKG2C+ NK cells and that of CMV-CTLs at different time points before and after CMV infection (C) or overall (D). Data are expressed as the mean and SEM. *, $p<0.05$.

Supplementary Figure 4

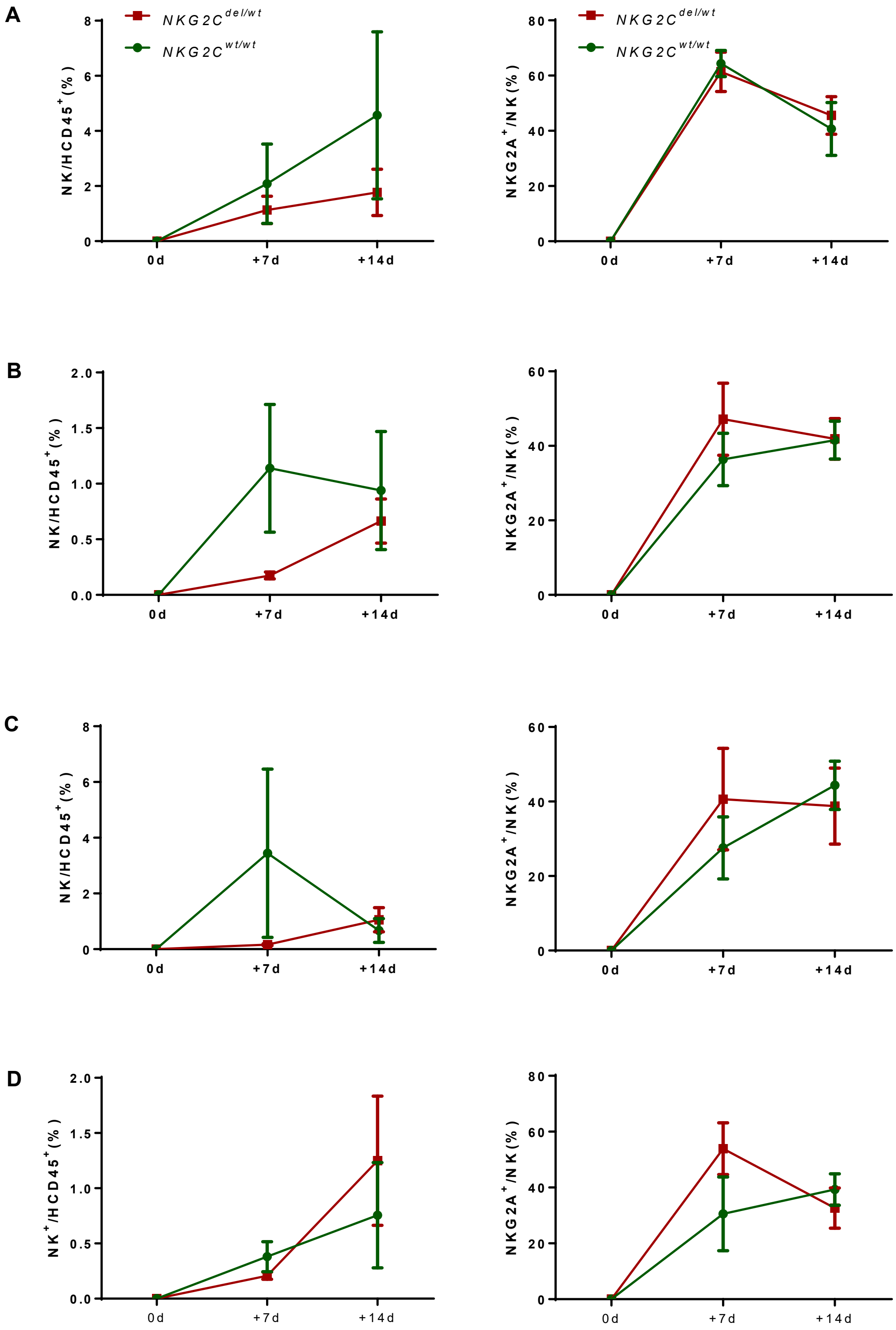

Supplementary Figure 4. The quantity of NK cell subsets in a humanized mouse model after NK cell infusion. The values of NK/HCD45^{+}(%) and NKG2A^{+}/NK(%) in the PB (A), spleen (B), liver (C) and lung (D) in the *NKG2C*$^{wt/wt}$ group and *NKG2C*$^{del/wt}$ group at days 0, 7 and 14. Data are expressed as the mean and SEM..

Supplementary Figure 5

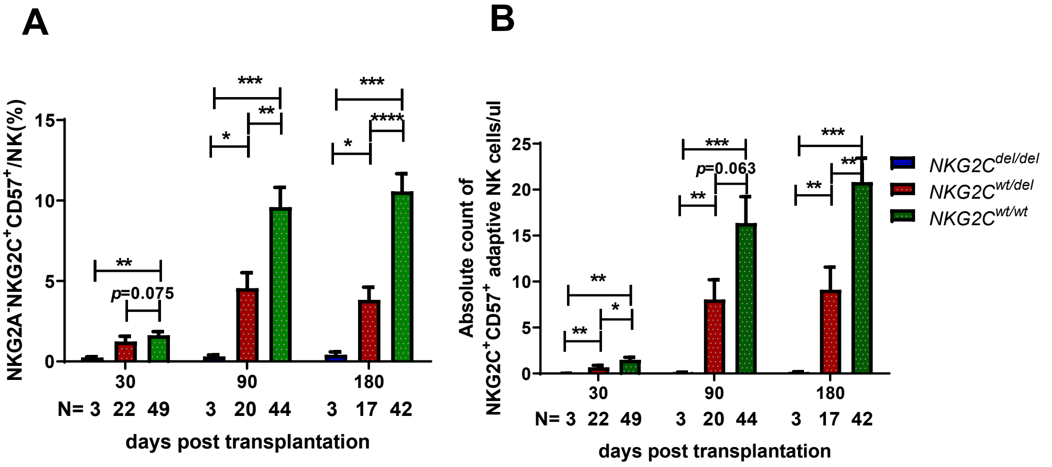

Supplementary Figure 5. Quantitative reconstitution of CD57+NKG2C+ adaptive NK cells in different NKG2C-genotype groups. The percentage (A) and absolute count (B) of NKG2A-CD57+NKG2C+ NK cells in the *NKG2C*$^{del/del}$, *NKG2C*$^{wt/del}$ and *NKG2C*$^{wt/wt}$ groups at days 30, 90, and 180 after allo-HSCT. Data are expressed as the mean and SEM. *, $p<0.05$, **, $p<0.01$, ***, $p<0.001$, ****, $p<0.0001$.

Supplementary Figure 6

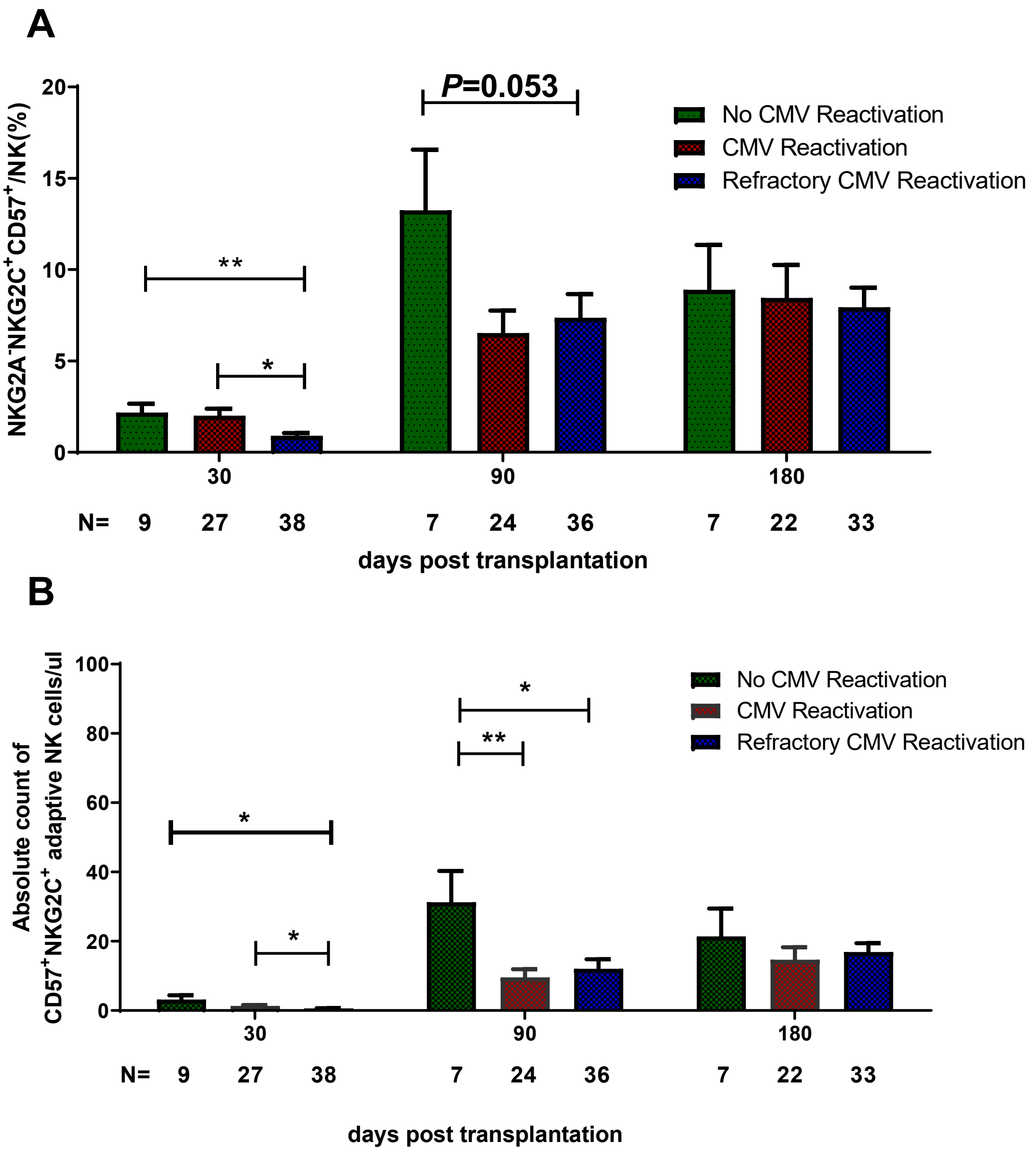

Supplementary Figure 6. Patients with refractory CMV reactivation showed poor quantitative reconstitution of CD57+NKG2C^{+} adaptive NK cells at day 30 after allo-HSCT. (A) The percentage of NKG2A^{-}CD57+NKG2C^{+} NK cells in the no-CMV-reactivation, CMV-reactivation, and refractory-CMV-reactivation groups at days 30, 90, and 180 after allo-HSCT. (B) The absolute count of CD57+NKG2C^{+} NK cells in the no-CMV-reactivation, CMV-reactivation, and refractory-CMV-reactivation groups at days 30, 90, and 180 after allo-HSCT. Data are expressed as the mean and SEM. *, $p<0.05$, **, $p<0.01$.

Supplementary Figure 7

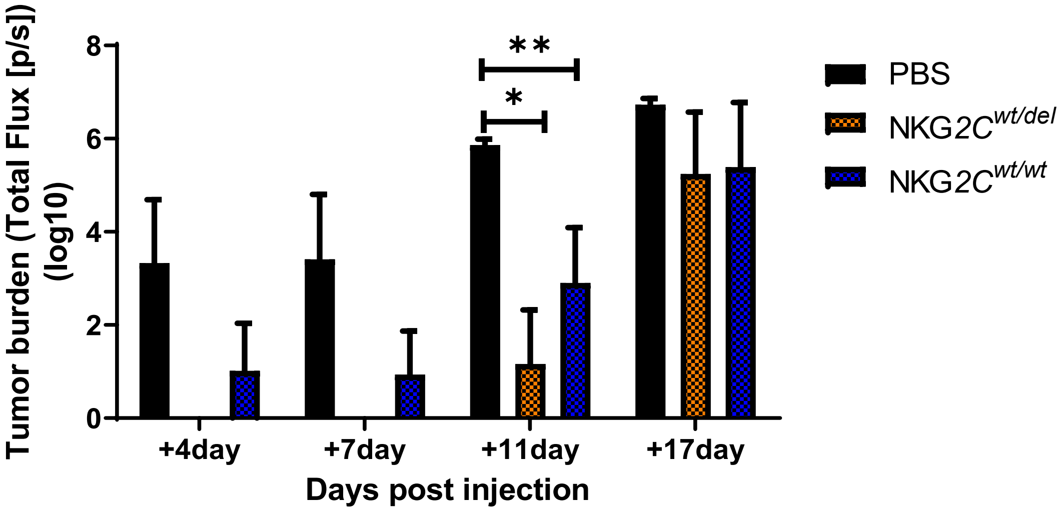

Supplementary Figure 7. The tumor burden of the PBS, *NKG2C*$^{del/wt}$ and *NKG2C*$^{wt/wt}$ groups at days 4, 7, 11 and 17 after K562-luc leukemia cell injection.

Supplementary Figure 8

A

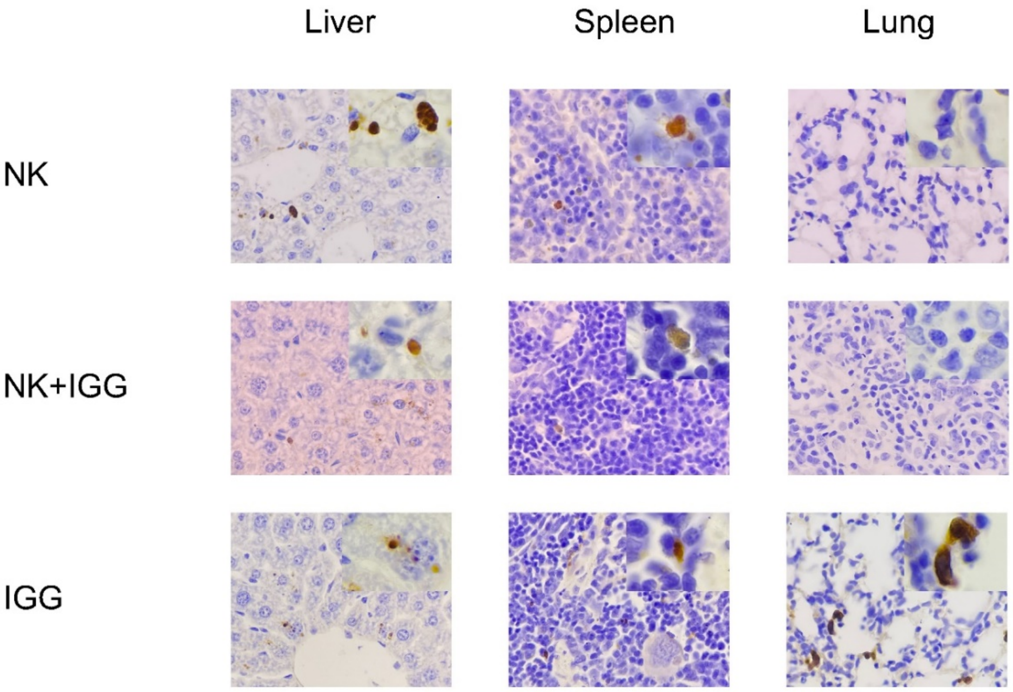

B

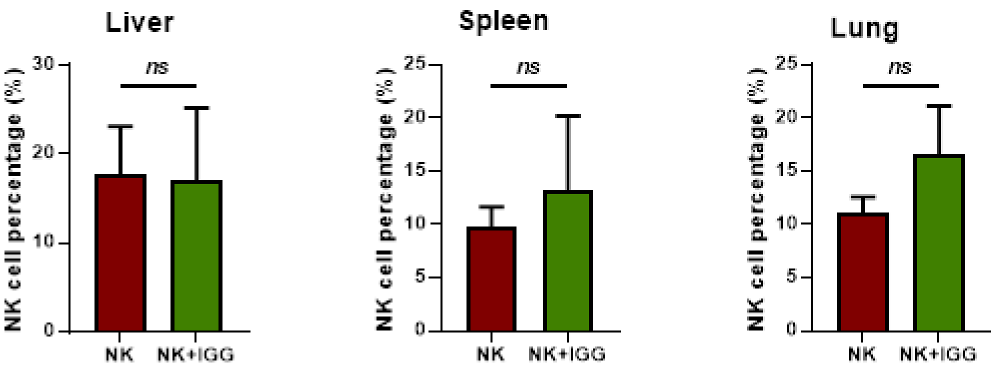

Supplementary Figure 8. CMV-IgG showed no effect on enhanced CMV clearance by 'adaptive' NK cells. At day 14 post *NKG2C*$^{del/wt}$ NK infusion, (A) mice in the IgG group remained positive for CMV DNA in the spleen, liver and lung. Mice in the NK plus IgG injection group showed CMV clearance only in the lung (original magnification ×40 and ×100). (B) NK cell percentages in the liver, spleen and lung of mice with only NK cell infusion or with both NK cell and IgG injection. No fewer than 5 mice were evaluated in each group.

Supplementary Table 1. Patient and donor characteristics the second case-paired prospective cohort

Characteristics	*NKG2C*$^{del/del}$	*NKG2C*$^{del/wt}$	*NKG2C*$^{wt/wt}$	*P* value
No. of patient	24	96	96	
Patient median age (range), years	34.5(11-63)	30(7-64)	30.5(9-63)	0.289
Patient sex, male, No.(%)	15(62.5%)	61(63.5%)	58(60.4%)	0.904
Donor median age (range), years	38(12-59)	41(9-64)	41.5(11-63)	0.340
Donor sex, male, No.(%)	15(62.5%)	63(65.6%)	65(67.7%)	0.879
Donor-recipient gender, No.(%)				0.984
Male to male	10(41.7%)	40(41.7%)	41(42.7%)	
Male to female	5(20.8%)	21(21.9%)	24(25.0%)	
Female to Male	6(25.0%)	21(21.9%)	17(17.7%)	
Female to Female	3(12.5%)	14(14.6%)	14(14.6%)	
Diagnosis, No.(%)				0.218
AML	14(58.3%)	73(76.0%)	68(0.8%)	
MDS	10(41.7%)	23(24.0%)	28(29.2%)	
Disease Status, high risk, No.(%)	0(0.0%)	0(0.0%)	0(0.0%)	1
HLA-A, HLA-B, HLA-DR mismatched grafts, No. (%)				0.103
1	2(8.3%)	2(2.1%)	1(1.0%)	
3	22(97.1%)	94(97.9%)	95(99.0%)	
ABO matched grafts, No.(%)				0.852
Matched	15(62.5%)	52(54.2%)	58(60.4%)	
Major mismatch	4(16.7%)	24(25.0%)	16(16.7%)	
Minor mismatch	4(16.7%)	15(15.6%)	18(18.8%)	
Bidirectional mismatch	1(4.2%)	5(5.2%)	4(4.2%)	
Donor-recipient CMV-IgG before transplantation, No.(%)				0.194
Donor(-) recipient(+)	2(8.3%)	7(7.3%)	2(2.1%)	
Donor(+) recipient(+)	22(91.7%)	89(92.7%)	94(97.9%)	
CMV reactivation, No.(%)	24(100.0%)	76(79.2%)	71(74.0%)	0.019
Refractory CMV reactivation, No.(%)	22(91.7%)	64(66.7%)	53(55.2%)	0.003
CMV reactivation episodes (median, range)	2(1-4)	1(0-5)	1(0-5)	0.078
CMV disease, No. (%)	0(0.0%)	3(3.1%)	0(0.0%)	0.149
aGVHD, No. (%)	13(54.2%)	49(51.0%)	56(58.3%)	0.597

Supplementary Table 2. Patient and donor characteristics in the first case-paired prospective cohort

Characteristics	*NKG2C*$^{del/wt}$	*NKG2C*$^{wt/wt}$	P value
No. of patient	22	22	
Patient median age (range), years	29.5(19-58)	32.5(15-63)	0.424
Patient sex, male, No.(%)	13(59.1%)	15(68.2%)	0.531
Donor median age (range), years	40(15-64)	29(12-60)	0.102
Donor sex, male, No.(%)	14(63.6%)	17(77.3%)	0.322
Donor-recipient gender, No.(%)			0.526
Male to male	9(40.9%)	12(54.5%)	
Male to female	5(22.7%)	5(22.7%)	
Female to Male	4(18.2%)	4(18.2%)	
Female to Female	4(18.2%)	1(4.5%)	
Diagnosis, No. (%)			0.792
AML	12(54.5%)	13(59.1%)	
CML	1(4.5%)	0(0.0%)	
ALL	5(22.7%)	5(22.7%)	
MDS	4(18.2%)	4(18.2%)	
HLA-A/B/DR mismatched grafts, No. (%)			0.549
1	0(0.0%)	1(4.5%)	
2	3(13.6%)	2(9.1%)	
3	19(86.4%)	19(86.4%)	
ABO matched grafts, No.(%)			0.241
Matched	15(62.8%)	14(63.6%)	
Major mismatch	5(22.7%)	3(13.6%)	
Minor mismatch	1(4.5%)	5(22.7%)	
Bidirectional mismatch	1(4.5%)	0(0.0%)	
CMV reactivation, No.(%)	21(95.5%)	19(86.4%)	0.294
Refractory CMV reactivation, No.(%)	14(63.3%)	10(44.5%)	0.226
aGVHD, No. (%)	12(54.5%)	12(54.5%)	1.000

Supplementary Table 3. Cox regression analysis of CMV infection risk in the second case-pair cohort

	CMV reactivation			refractory CMV reactivation		
	HR	95%CI	*P*	HR	95%CI	*P*
NKG2C genotype			0.045			0.004
$NKG2C^{wt/del}$ vs $NKG2C^{del/del}$	0.598	0.377-0.949	0.029	0.535	0.328-0.872	0.012
$NKG2C^{wt/wt}$ vs $NKG2C^{del/del}$	0.560	0.351-0.892	0.015	0.426	0.258-0.703	0.001
aGVHD	0.651	0.479-0.885	0.006	0.639	0.454-0.899	0.010

Supplementary Table 4. The donors' genotyping of IGG1, CD16, and HLA-E for humanized mice

Donor	IGHG	FCGR3A	HLA-E	NKG2C
1	CC (G1m3)	AA (CD16A-F/F)	01:01	wt/del
2	CC (G1m3)	AA (CD16A-F/F)	01:01	wt/wt
3	TC (G1m3/17)	AC (CD16A-V/F)	01:03	wt/del
4	TT (G1m17)	AA (CD16A-F/F)	01:01	wt/del
5	CC (G1m3)	AA (CD16A-F/F)	01:03	wt/wt
6	TT (G1m17)	AA (CD16A-F/F)	01:01	wt/wt

Reference

1. Moraru M, Black LE, Muntasell A, Portero F, Lopez-Botet M, Reyburn HT, et al. NK Cell and Ig Interplay in Defense against Herpes Simplex Virus Type 1: Epistatic Interaction of CD16A and IgG1 Allotypes of Variable Affinities Modulates Antibody-Dependent Cellular Cytotoxicity and Susceptibility to Clinical Reactivation. *J Immunol.* 2015;195(4):1676-84.
2. de Miranda BLM, Gelmini GF, Risti M, Hauer V, da Silva JS, Roxo V, et al. HLA-E genotyping and its relevance in kidney transplantation outcome. *HLA.* 2020;95(5):457-64.
3. Wagner B, Dührsen U, Hüttmann A, Nückel H, Michita RT, Rohn H, et al. Genetic Variants of the NKG2C/HLA-E Receptor-Ligand Axis Are Determinants of Progression-Free Survival and Therapy Outcome in Aggressive B-Cell Lymphoma. *Cancers.* 2020;12(11).
